# Spatiotemporal patterning of EpCAM is important for murine embryonic endo- and mesodermal differentiation

**DOI:** 10.1038/s41598-018-20131-8

**Published:** 2018-01-29

**Authors:** Sannia Sarrach, Yuanchi Huang, Sebastian Niedermeyer, Matthias Hachmeister, Laura Fischer, Sebastian Gille, Min Pan, Brigitte Mack, Gisela Kranz, Darko Libl, Juliane Merl-Pham, Stefanie M. Hauck, Elisa Paoluzzi Tomada, Matthias Kieslinger, Irmela Jeremias, Antonio Scialdone, Olivier Gires

**Affiliations:** 1 Department of Otorhinolaryngology, Head and Neck Surgery, Grosshadern Medical Center, Ludwig Maximilians University, Munich, Marchioninistr. 15, 81377 Munich, Germany; 2 Department of Orthopedics, Spine Surgery, Baoji Central Hospital, JiangTan Road. 8, 721008 Baoji, China; 3 Research Unit Protein Science, Helmholtz Zentrum München, Deutsches Forschungszentrum für Gesundheit und Umwelt, Ingolstädter Landstraße 1, 85764 Neuherberg, Germany; 4 Department of Gene Vectors, Helmholtz Zentrum München, German Center for Environmental Health (HMGU), Marchioninistr. 25, 81377 Munich, Germany; 5 Institute of Medical Genetics, Medical University of Vienna, Austria, Währingerstr. 10, 1090 Vienna, Austria; 6 German Consortium for Translational Cancer Research (DKTK), Partnering Site, Munich, 81337 Munich, Germany; 7 Department of Pediatrics, Dr. von Hauner Children’s Hospital, Ludwig Maximilians University, München, 80337 Munich, Germany; 8 Institute of Epigenetics and Stem Cells, Helmholtz Zentrum München, Deutsches Forschungszentrum für Gesundheit und Umwelt, Marchioninistr. 25, 81377 Munich, Germany; 9 Institute of Functional Epigenetics, Helmholtz Zentrum München, Deutsches Forschungszentrum für Gesundheit und Umwelt, Ingolstädter Landstraße 1, 85764 Neuherberg, Germany; 10 Institute of Computational Biology, Helmholtz Zentrum München, Deutsches Forschungszentrum für Gesundheit und Umwelt, Ingolstädter Landstraße 1, 85764 Neuherberg, Germany

## Abstract

Epithelial cell adhesion molecule EpCAM is expressed in pluripotent embryonic stem cells (ESC) *in vitro*, but is repressed in differentiated cells, except epithelia and carcinomas. Molecular functions of EpCAM, possibly imposing such repression, were primarily studied in malignant cells and might not apply to non-pathologic differentiation. Here, we comprehensively describe timing and rationale for EpCAM regulation in early murine gastrulation and ESC differentiation using single cell RNA-sequencing datasets, *in vivo* and *in vitro* models including CRISPR-Cas9-engineered ESC-mutants. We demonstrate expression of EpCAM in inner cell mass, epiblast, primitive/visceral endoderm, and strict repression in the most primitive, nascent Flk1^+^ mesoderm progenitors at E7.0. Selective expression of EpCAM was confirmed at mid-gestation and perinatal stages. The rationale for strict patterning was studied in ESC differentiation. Gain/loss-of-function demonstrated supportive functions of EpCAM in achieving full pluripotency and guided endodermal differentiation, but repressive functions in mesodermal differentiation as exemplified with cardiomyocyte formation. We further identified embryonic Ras (ERas) as novel EpCAM interactor of EpCAM and an EpCAM/ERas/AKT axis that is instrumental in differentiation regulation. Hence, spatiotemporal patterning of EpCAM at the onset of gastrulation, resulting in early segregation of interdependent EpCAM^+^ endodermal and EpCAM^−^/vimentin^+^ mesodermal clusters represents a novel regulatory feature during ESC differentiation.

## Introduction

Epithelial cell adhesion molecule EpCAM was originally described as a cell surface antigen highly expressed in human carcinomas^1^. Today, we know that EpCAM is present as a heart-shaped cis-dimer at the cell surface^2^, and that it has a broader but nevertheless sharply restricted expression pattern in undifferentiated pluripotent embryonic stem cells (ESC)^3–5^, hepatic, pancreatic epithelial and other endodermal progenitor cells^6–8^, epithelium^9^, carcinoma and cancer stem cells^10,11^. Other fully differentiated cell types entirely lack expression of EpCAM. This selective expression implies substantial dynamics and tight control of EpCAM throughout differentiation of ESC into specified cell types. Relief from this tight regulation are known from malignant transformation, where EpCAM is *de novo* expressed or up-regulated in carcinomas^10–12^.

Precise timing and rationale for this selective expression pattern in differentiation remains largely elusive. Molecular functions of EpCAM that could be the cause of this restrictive expression have primarily been studied in cancer cells and might thus not be entirely transferred to non-pathologic differentiation processes. In cancer cells, EpCAM regulates cell-cell adhesion^13,14^ and proliferation^15,16^, the later based on regulated intramembrane proteolysis (RIP) and nuclear translocation of the intracellular domain EpICD^17,18^. RIP-dependent processing of EpCAM was also reported in murine and human ESC^3,19^. In human and porcine ESC, EpICD supports pluripotency through activation of promoters of the reprogramming factors Sox2, Oct3/4 and Nanog^3,20,21^. Additionally, EpEX/EpCAM is, together with Oct3/4 or KLF4, sufficient to generate induced pluripotent stem cells in the human system^22^.

Genetic knockout of *EPCAM* in mice was initially reported to induce embryonic lethality^23^. Subsequent knockout strains disclosed a role in intestinal epithelium integrity through regulation of tight or *adherens* junctions, resulting in severe post-natal bleeding and death^24,25^. Both mouse models mimicked human congenital tufting enteropathy that results in life-threatening watery diarrhoea owing to the loss of intestinal cell surface expression of EpCAM^26^. Genetic silencing of EpCAM further confirmed its role in tight junction formation, based on functions in the actomyosin network homoestasis and control of cortical tension at tricellular contacts^27^. Further implications of EpCAM in differentiation were related to motility and migration of skin Langerhans cells in mice^28^ and morphogenic movements during gastrulation in *Xenopus laevis*^29,30^ and zebrafish^31^. Hence, regulation of EpCAM expression and function appear instrumental in differentiation, but knowledge on the actual timing of regulation and on the impact of retention or loss on cell fates at early stages of embryogenesis remains fragmentary.

Here, we have addressed timing and the basic rationale for the tight regulation of EpCAM during murine development. We describe a spatiotemporal patterning of EpCAM in initiating gastrulation and throughout embryonic differentiation that is important for proper differentiation of ESC into endo- and mesodermal cells, with retention of EpCAM in the first and complete loss in the later cells. We further identified embryonic Ras as a novel interaction partner of EpCAM that is instrumental during differentiation regulation. Thus, spatiotemporal patterning of EpCAM is an early and important regulatory feature of differentiating ESC.

## Results

### EpCAM expression in early murine gastrulation

**Figure 1:**
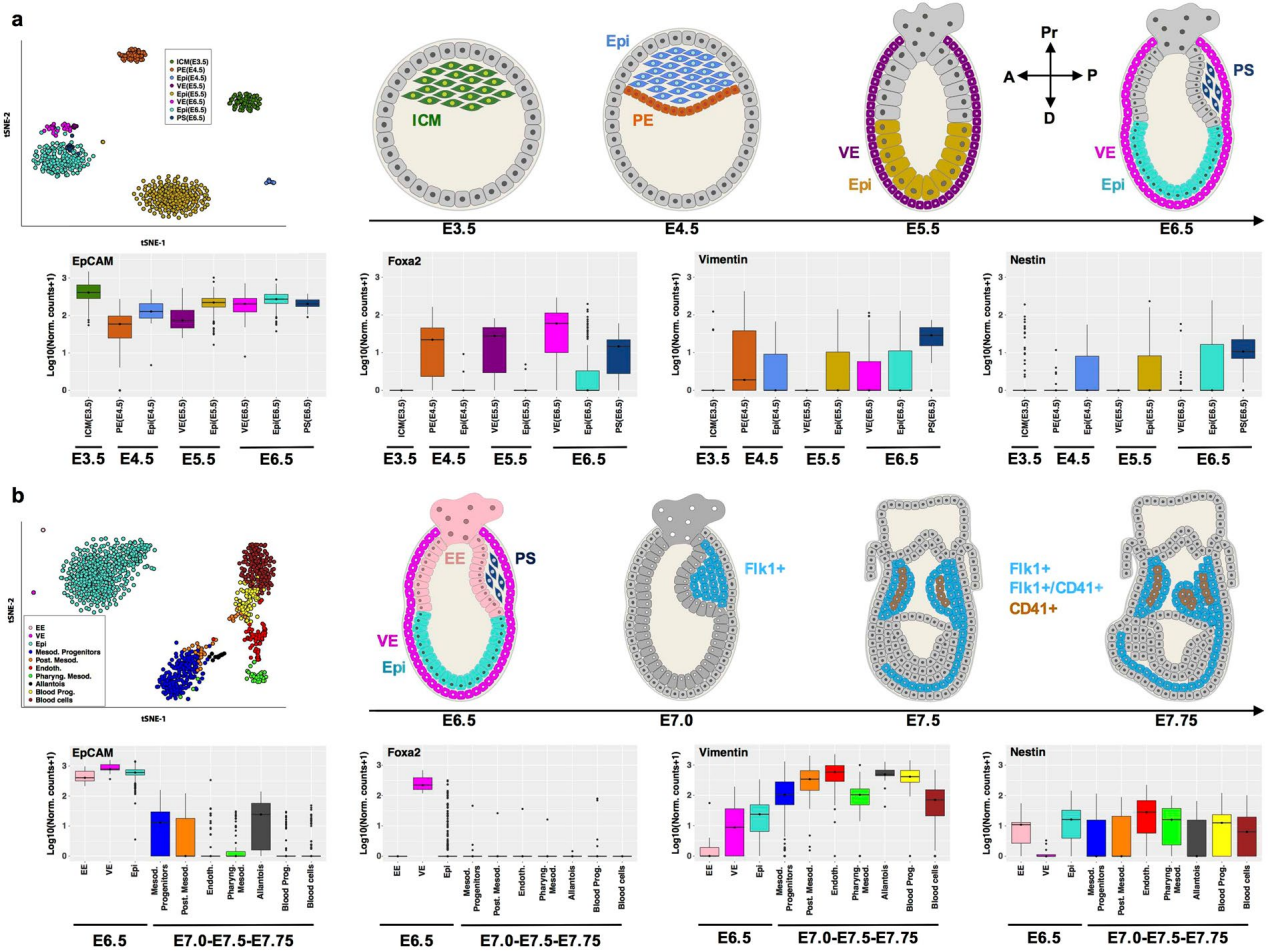
EpCAM expression in early- and mid-gastrulation. **(a,b)** Transcript levels of EpCAM, Foxa2, vimentin and nestin were assessed from RNA-sequencing datasets generated from single cells at day E3.5–6.5^32^ (**a**) and E6.5–7.75^33^ (**b**) of murine embryos. We show a visualization of the datasets with t-distributed stochastic neighbor embedding^67^ (upper left panels), by highlighting the clusters defined in the original publications^32,33^. The approximate localization of cells included in each dataset is depicted in schemes for each time point (A: anterior; P: posterior; Pr: proximal; D: distal). Transcript levels are depicted as box-whisker plots with log_10_ normalized counts (adding a pseudocount of 1), and colored according to cell type (lower panels). See Methods for additional details.

In order to gain insight in the regulation pattern of EpCAM during early embryonic development, we re-analyzed two previously published single cell RNA-sequencing datasets of mouse embryos^32,33^, spanning a developmental time going from blastocyst (E3.5) to head fold (E7.75) and including several embryonic and extra-embryonic lineages (see Fig. 1).

EpCAM mRNA expression was highest in cells of the inner cell mass (ICM) that comprises ESC, was slightly reduced but remaining high in the epiblast at E4.5, E5.5 and E6.5, and was sustained at high levels throughout early endodermal differentiation, including primitive endoderm (E4.5), and various stages of visceral endoderm (E6.5–6.75), and in the primitive streak (Fig. 1a).

EpCAM was co-expressed with the endodermal marker Foxa2 in cells of the primitive (E4.5) and visceral (E5.5–6.5) endoderm, and in cells of the forming primitive streak (E6.5) (Fig. 1a and Supplementary Figure 1). Foxa2 mRNA expression was absent in the inner cell mass and early epiblast cells, and was increased in single cells of E6.5 epiblast. Expression of the mesodermal and ectodermal markers vimentin and nestin, respectively, was detectable but low in all stages of early embryonic development. Co-expression of EpCAM, vimentin, and nestin was mostly detected in cells of the forming primitive streak, where vimentin and nestin were expressed at comparably low levels (Fig. 1a and Supplementary Figure 1).

Next, expression of EpCAM mRNA was analyzed in a second dataset including single cells isolated from the epiblast (E6.5), and from Flk1^+^ (E7.0), Flk1^+^/CD41^+^ and Flk1^−^/CD41^+^ progeny (E7.5–7.75)^33^. As described in the original publication, ten clusters could be identified in this dataset, which comprise several mesodermal lineages and embryonic blood cells (Fig. 1b). EpCAM mRNA expression was high in cells of the extraembryonic ectoderm, the visceral endoderm and the epiblast at E6.5 (Fig. 1b), thus confirming the aforementioned expression pattern. Nascent mesodermal progenitors and posterior mesoderm cells were characterized by a substantial ~100-fold and ~500-fold down-regulation of EpCAM expression, respectively (Fig. 1b). Further differentiated mesodermal cells including endothelium, pharyngeal mesoderm, blood progenitors and blood cells, were largely devoid of EpCAM mRNA expression, except cells of the allantois, which expressed residual levels comparable to nascent mesodermal progenitors (Fig. 1b). EpCAM was co-expressed with Foxa2 in cells of the visceral endoderm (Fig. 1b and Supplementary Figure 1), while the expression of mesodermal marker vimentin displayed a complementary pattern of expression with respect to EpCAM (Fig. 1b). Indeed, vimentin expression was expectedly induced in nascent mesodermal progenitors and was sustained in their progeny. Ectodermal marker nestin was expressed at low levels in all differentiation stages analyzed and, thus, showed little co-expression with EpCAM (Fig. 1b and Supplementary Figure 1).

Overall, EpCAM expression was associated with pluripotent and endodermal cells throughout early embryonic development, and was negatively correlated with mesodermal marker vimentin owing to an early repression in nascent mesoderm.

### EpCAM patterning from mid-gestation to perinatal embryonic development

**Figure 2:**
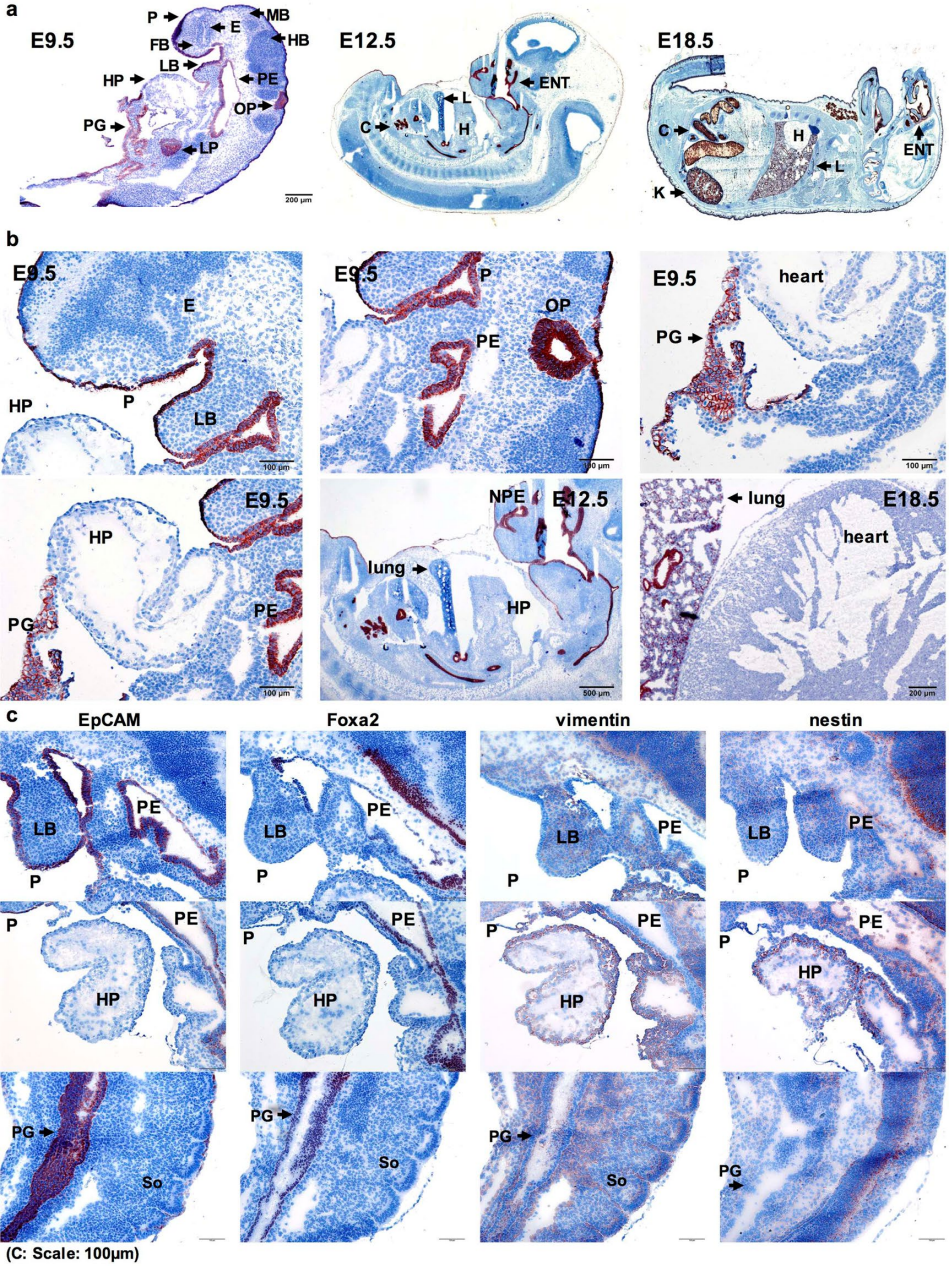
EpCAM expression in mid-gestation to perinatal mouse embryos. (**a**) EpCAM expression was detected by immunohistochemistry staining (brown) in sections of cryo-conserved C57BL-6 embryos at gestation day E9.5 (scale bar represents 200 µm), E12.5 and E18.5, as indicated. Cytoplasm and nucleus were counterstained with H&E. **(b)** Magnification of EpCAM expression in embryos at E9.5 (scale bar represents 100 µm), E12.5 (scale bar represents 200 µm) and E18.5 (scale bar represents 200 µm). **(c)** EpCAM, Foxa2, vimentin and nestin protein expression is depicted in consecutive sections of E9.5 embryos (scale bars represent 100 µm). C colon; E eye; ENT ear nose and throat area; FB fore-brain; H heart; HB hind-brain; HP heart primordium; K kidney; L lung; LB limb bud; LP liver primordium; MB mid-brain; OP otic pit; P periderm; PE pharyngeal epithelium; PG primitive gut; So somite.

Based on RNA-sequencing data demonstrating an early patterning of EpCAM expression in endo- versus mesodermal cells, we analyzed the expression of EpCAM protein in C57BL-6 mouse embryos from mid-gestation (E9.5–12.5) to perinatal time point E18.5. At gestation day E9.5, EpCAM was detectable in the periderm (P) enveloping the embryo, epithelia of pharyngeal primordium (PE), primitive gut (PG), otic pit (OP), and liver primordium (LP), but was lacking in meso- and ectodermal cells including heart, fore-, mid- and hindbrain (HP, FB, MB, HB) (Fig. 2a–c and Supplementary Figure 2). Expression of EpCAM and vimentin was mutually exclusive in gut and liver endoderm versus cardiac mesoderm (Fig. 2a,b and Supplementary Figure 2). Similar expression of EpCAM in endodermal structures (*e.g*. lungs, colon epithelia), but a lack in mesodermal structures (*e.g*. heart) was observed at later embryonic E12.5 and E18.5 stages (Fig. 2a,b and Supplementary Figure 2).

Selective expression of EpCAM was associated with the endodermal marker Foxa2 in the pharyngeal area, and primitive gut, while both were lacking in vimentin^+^ heart primordium. EpCAM, but not Foxa2, was expressed in the embryonic periderm, which derives from surface ectoderm (itself a derivative of the EpCAM^+^ epiblast) (Fig. 2c). At perinatal stage E18.5, EpCAM was present in epidermis, hair follicles, epithelial lining and root of tongue, larynx, pharynx, auditory channel, salivary glands, liver, lung, epithelial cells of kidney tubules, and colon (Supplementary Figure 2c).

Hence, EpCAM protein expression from mid-gestation to perinatal embryos confirmed RNA-sequencing expression data of early embryonic developmental stages. In particular, EpCAM displayed partially overlapping expression pattern with Foxa2 in endodermal tissues, but was mutually exclusive with vimentin in mesoderm.

### EpCAM patterning in early 3D-differentiation of ESC

**Figure 3:**
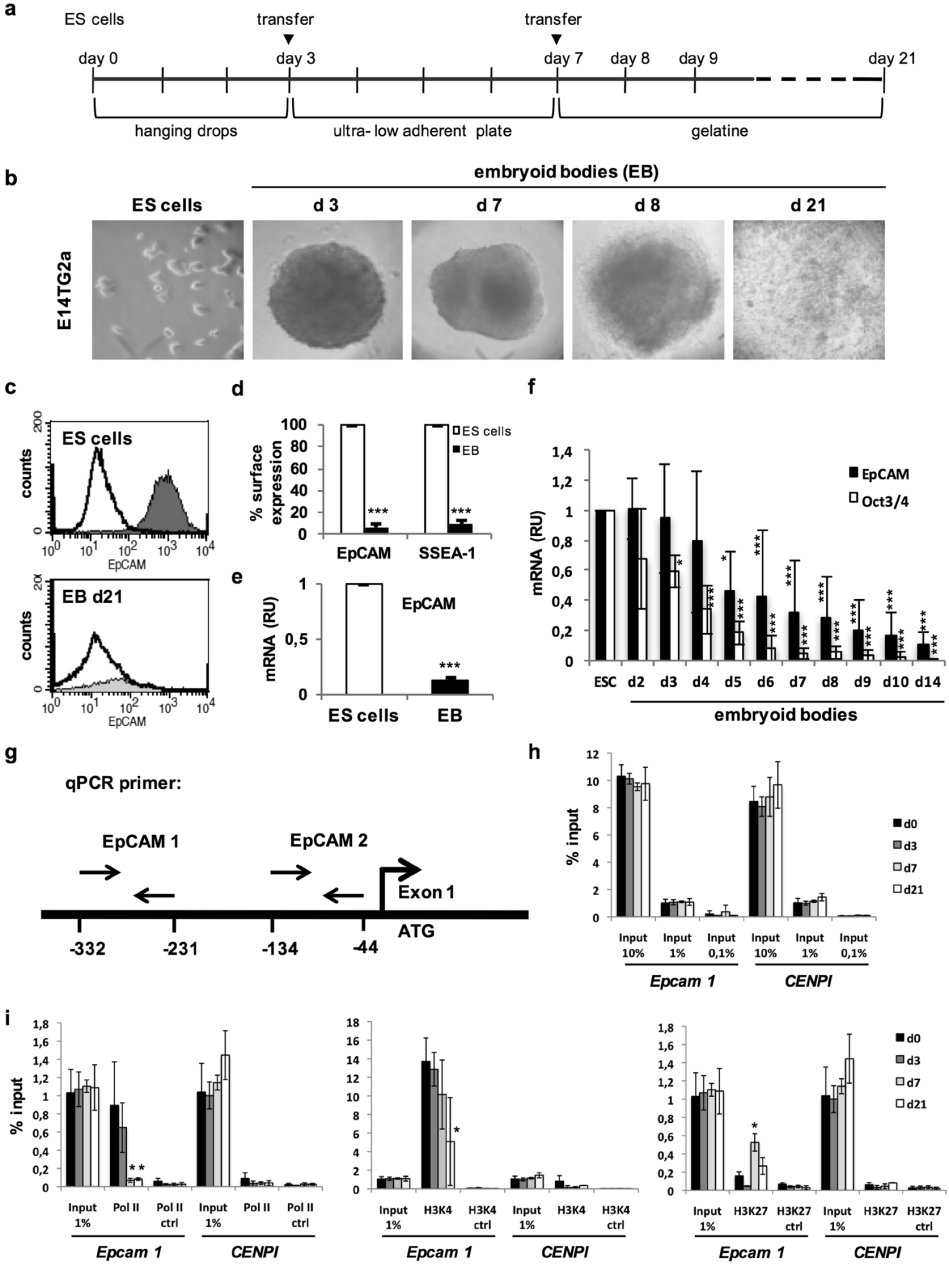
EpCAM expression in differentiating ESC. **(a)** Schematic depiction of the timeline of EB formation. **(b)** Representative pictures of E14TG2α ESC in 2D culture (ES cells) and embryoid bodies (EB) at the indicated time points of spontaneous 3D-differentiation. **(c)** Representative FACS histogram of EpCAM expression in pluripotent E14TG2α ESC and EB at differentiation day 21. **(d)** Mean EpCAM and SSEA1 cell surface expression measured by FACS analysis in pluripotent E14TG2α ESC and EB (d21) (n = 3 independent experiments). **(e)** Mean EpCAM mRNA expression measured by quantitative PCR in pluripotent E14TG2α ESC and differentiated EB (day 21) (n = 3 independent experiments). (**f)** Kinetic of EpCAM and Oct3/4 mean mRNA expression measured by quantitative PCR in pluripotent and differentiating ESC (n = 3 independent experiments). **(g)** Schematic depiction of primer pairs relative to transcription start site (ATG) of *EPCAM*. **(h)** Time and concentration kinetics of input control at *EPCAM* promoter and *CENPI* locus from chromatin-IP samples. (n = 3 independent experiments). **(i)** Chromatin-IP (ChIP) of polymerase II (Pol II), H3K4 and H3K27 at *EPCAM* promoter and *CENPI* locus (n = 3 independent experiments). Shown are mean values of quantitative PCR amplification of the region of the EPCAM promoter after ChIP with the indicated specific antibodies. (n = 3 independent experiments). Mean ± SEM; Student’s T-test (n = 2 groups) or One-Way ANOVA (n ≥ 3 groups); p < 0.05, **p < 0.01, ***p < 0.001.

We made use of a hanging-drop 3D-differentiation model to generate embryoid bodies (EB) from E14TG2α ESC (Fig. 3a,b), which closely mimics embryogenesis *in vitro* and allows genetic manipulations^34^.

Down-regulation of cell surface expression of EpCAM and pluripotency marker SSEA-1 by more than 90% was observed in differentiated EB (day 21) compared to pluripotent ESC (Fig. 3c,d). Loss of EpCAM mRNA by 90% (Fig. 3e) was progressive and slightly delayed compared to core reprogramming factor Oct3/4 (Fig. 3f). Substantial down-regulation of EpCAM expression during 3D-differentiation was confirmed in the Bruce4 ESC line, which expresses similar levels of EpCAM under pluripotency conditions (Supplementary Figure 3a). Upon 3D-differentiation, Bruce4 ESC substantially down-regulated EpCAM and SSEA-1 expression at the cell surface and EpCAM and Oct3/4 at the mRNA level (Supplementary Figure 3b–d).

Chromatin immunoprecipitation (ChIP) experiments displayed enrichment of polymerase 2 (Pol II) and activating trimethylation of histone 3 at lysine 4 (H3K4) at two sites within the promoter of the murine *EPCAM* gene in pluripotent ESC (Fig. 3g,i). Control amplifications at the *CenpI* locus did not show any enrichment for Pol II, H3K4, or H3K27 and input controls revealed comparable levels (Fig. 3h,i). Upon differentiation, Pol II binding and H3K4 trimethylation progressively decreased, while inhibitory trimethylation of histone 3 at lysine 27 (H3K27) increased (Fig. 3i). Data mining of ChIP results confirmed Pol II and methylation pattern at the *EpCAM* promoter in undifferentiated Bruce4 ESC. In heart cells of eight weeks old mice, the *EPCAM* promoter lacked Pol II, with weak H3K4 and strong H3K27 trimethylation. Comparable chromatin silencing was observed in liver, brain, and spleen, while kidney and thymus displayed presence of all three marks, reflecting EpCAM heterogeneity in these organs (Supplementary 4). Thus, EB differentiation mimics expression dynamics of EpCAM observed in early embryos, with a strong reduction of protein expression and of transcription due to epigenetic control.

### Early segregation of EpCAM^+^ and EpCAM^−^ cell clusters of differentiating ESC

**Figure 4:**
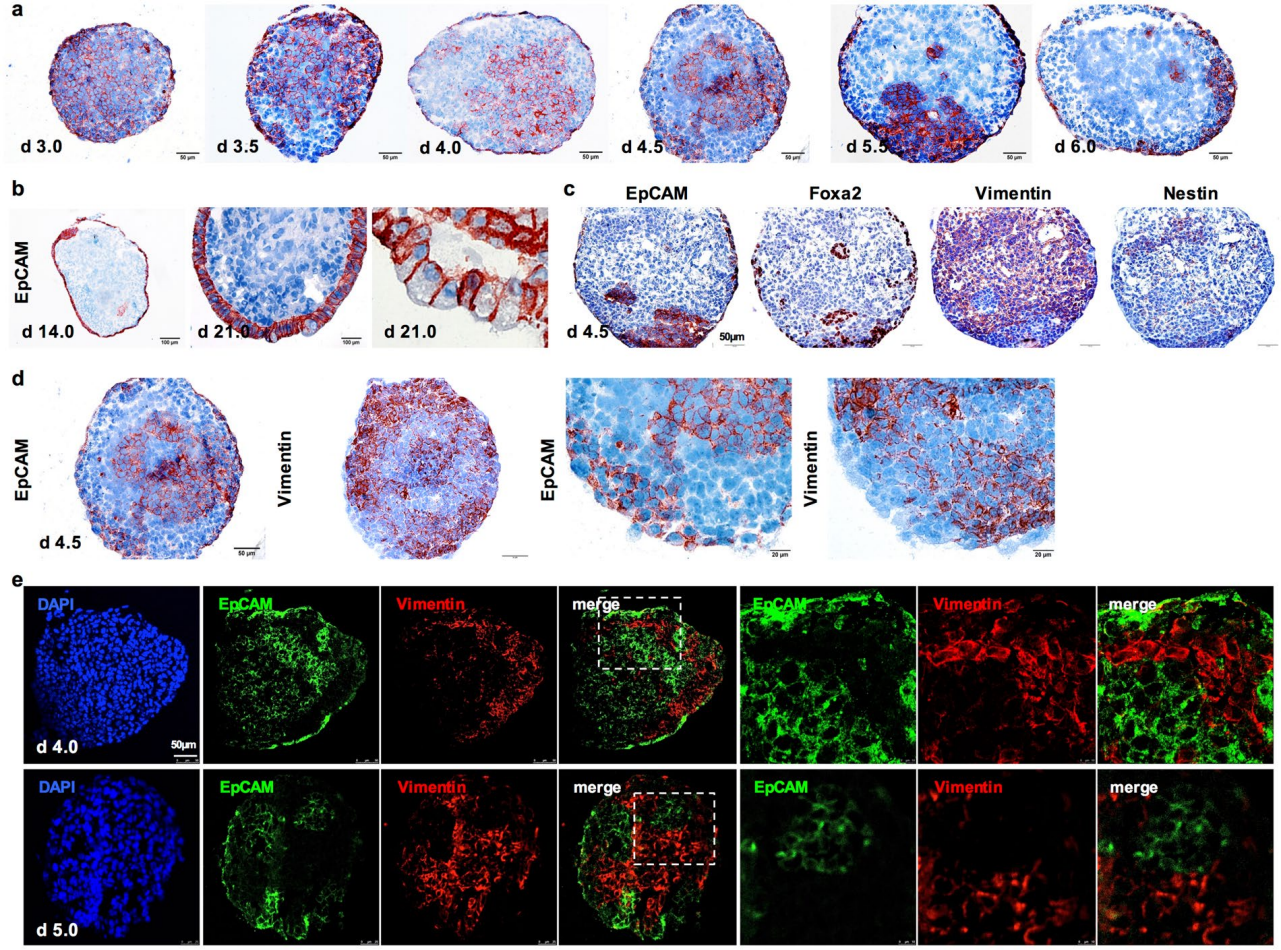
EpCAM patterning in differentiating ESC. (**a**) EpCAM expression during 3D-differentiation of ESC is depicted through immunohistochemistry staining in E14TG2α EB (d3–6; scale bars represent 50 µm). (**b**) Representative EpCAM staining in d14 and d21 EB showing basolateral staining in differentiated epithelia. (**c**) EpCAM, Foxa2 (endoderm), vimentin (mesoderm) and nestin (ectoderm) expression in consecutive sections of EB at d4 (scale bars represent 100 µm). (**d**) EpCAM and vimentin expression in consecutive sections of EB at d4 (200x and 400x magnification; scale bars represent 50 and 20 µm). (**e**) EpCAM and vimentin expression in EB at day 4 (upper) and 5 (lower) depicted as double-immunofluorescence staining (EpCAM: green; vimentin: red). Left panels comprise full overviews of EBs; right panels represent magnifications of the area demarked in the doted squares. Shown are representative pictures from ≥3 independent experiments with multiple EB each (scale bar represents 50 µm). Staining of the indicated markers is depicted in brown. Cytoplasm and nucleus were counterstained with H&E, except for immunofluorescence staining in (**e**).

Next, we analyzed the expression pattern of EpCAM during 3D-differentiation in EB at the cellular level. In differentiating EB of E14TG2α ESC, loss of EpCAM expression and segregation of EpCAM^+^ and EpCAM^−^ clusters from day 3.5 onwards resulted in spatiotemporal patterning of EpCAM (Fig. 4a). Typically, a layered margin of flattened visceral endoderm cells expressed EpCAM from day 4.0 onwards, while progressive loss of EpCAM was observed in the remaining EB, resulting in a majority of cells entirely devoid of EpCAM after day 6.0 of differentiation (Fig. 4a). Over time, EpCAM^+^ cells were further restricted and differentiated to prismatic epithelium characterized by a basolateral expression pattern of EpCAM (Fig. 4b). Similar spatiotemporal patterning was observed in EB generated from Bruce4 ESC, although with a slightly delayed time course, which reflected differences in proliferation rates of both cell lines (Supplementary Figure 3e).

Early in ESC differentiation (EB d4.5), EpCAM partially overlapped with Foxa2 (prominently in cells of the visceral endoderm) and was mutually exclusive to vimentin (Fig. 4c,d). Neither strict negative nor positive correlation was observed between EpCAM and nestin (Fig. 4c). Mutually exclusive expression of EpCAM and vimentin was further confirmed with immunofluorescence double-staining of EB at day 4 and 5 (Fig. 4e). Immunofluorescence double-staining of EpCAM with Foxa2 validated the presence of EpCAM^+^/Foxa2^+^ visceral endoderm cells at the edge of EBs (Supplementary Figure 5a), whereas no obvious correlation was observed upon double-staining of EpCAM and nestin (Supplementary Figure 5b). Hence, 3D-differentiation of ESC in EB recapitulated EpCAM^+^ and EpCAM^−^/vimentin^+^ cell cluster segregation observed *in vivo*.

### EpCAM regulation in pluripotency and endodermal differentiation

**Figure 5:**
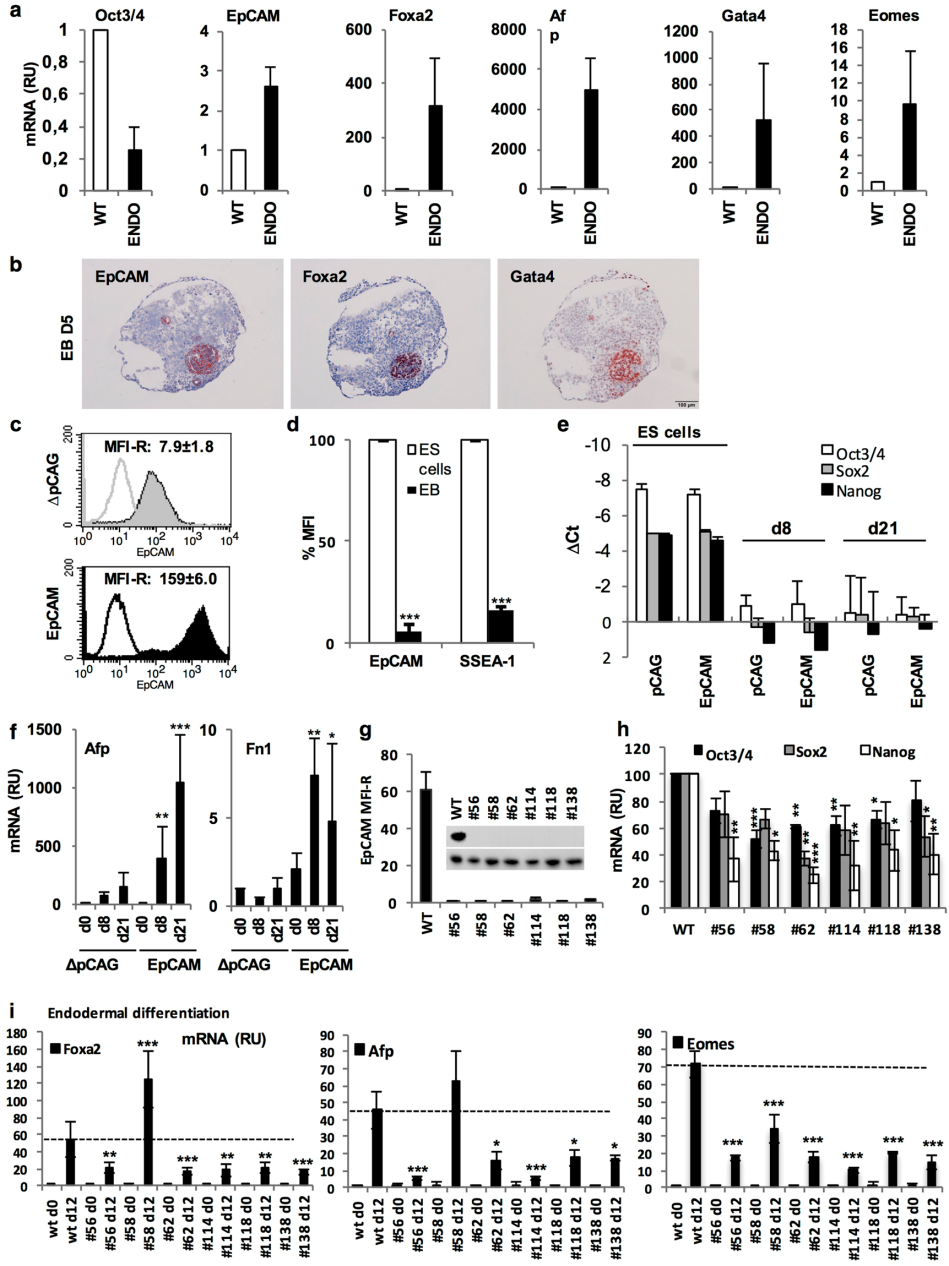
EpCAM impact on endodermal differentiation. (**a**) E14TG2α ESC were subjected to endodermal differentiation upon treatment with retinoic acid (RA) and basic fibroblast growth factor (bFGF) as described in the Methods section. Oct3/4, EpCAM, Foxa2, Gata4, Eomes, and Afp mRNA levels were assessed by quantitative PCR in pluripotent (WT) and differentiated cells. (n = 2 independent experiments with duplicates). (**b**) EpCAM, Foxa2 and Gata4 expression in EB (d5). Shown are representative immunohistochemistry staining of EB from two independent experiments with multiple EB each. (**c**) Representative diagram of cell surface expression of EpCAM in pCAG and EpCAM transfectants measured by FACS (n = 3 independent experiments). **(d)** Shown are mean cell surface expression of EpCAM and SSEA-1 in EpCAM transfectants of pluripotent ESC and differentiated EB at day 21 (n = 3 independent experiments). (**e**) *Oct3/4*, *SOX2* and *NANOG* mRNA expression was measured by quantitative PCR in pluripotent (ES cells) and differentiated transfectants at the indicated time points (n = 3 independent experiments). (**f**) Afp and Fn1 mRNA expression was measured by quantitative PCR in pluripotent and differentiated pCAG and EpCAM-expressing E14TG2α ESC at the indicated time points (n = 3 independent experiments). **(g)** Cell surface EpCAM expression was measured by FACS and in cell lysates of wild-type and *EPCAM* knockout clones under pluripotency conditions (n = 3 independent experiments). **(h)** Oct3/4, Sox2 and Nanog mRNA expression was measured by quantitative PCR in wild-type and *EpCAM* knockout clones under pluripotency conditions (n = 3 independent experiments). **(i)** Foxa2, Afp and Eomes mRNA expression was measured by quantitative PCR in wild-type and *EpCAM* knockout clones after endodermal differentiation upon treatment with RA and bFGF at day 5 as described in Methods section (n = 3 independent experiments). Mean ± SEM; Student’s T-test (n = 2 groups) or One-Way ANOVA (n ≥ 3 groups); p < 0.05, **p < 0.01, ***p < 0.001.

In the following, we addressed the rationale for the observed association of EpCAM with early endoderm and repression in mesoderm. Endodermal differentiation of ESC was induced upon treatment with basic fibroblast growth factor (bFGF) and retinoic acid (RA) as described^35^. Differentiation was confirmed by 80% reduction of Oct3/4 and induction of Foxa2, Gata4, Eomes, and Afp (Fig. 5a). In contrast to a >90% reduction during spontaneous differentiation, EpCAM mRNA levels were enhanced by 2.6-fold compared to pluripotent ESC upon endodermal differentiation (Fig. 5a). Hence, EpCAM expression in spontaneous differentiation and fostered endodermal differentiation vary by a factor of >25-fold and was tightly co-expressed with Foxa2^+^/Gata4^+^ in endodermal clusters (Fig. 5b).

In order to assess its contribution to pluripotency and differentiation, EpCAM was ectopically expressed from the CMV early promoter in E14TG2α ESC. The CMV promoter was chosen based on its capacity to drive expression of genes in ESC with a retained ability to be (down)-regulated during differentiation^36^. Over-expression of EpCAM at the surface of pluripotent ESC by a factor of 20-fold was feasible (Fig. 5c and Supplementary Figure 6a). However, similar to endogenous EpCAM protein, ectopic expression was reduced by 90% during spontaneous 3D-differentiation compared to the pluripotent state of over-expression, suggesting a necessity for EpCAM reduction during differentiation. It must be noted that EpCAM transfectants remained with EpCAM levels superior than wild-type ESC after differentiation, owing to the initial over-expression (Supplementary Figure 6b). Unlike endogenous EpCAM mRNA levels, which were consistently reduced by 90% upon spontaneous differentiation, EpCAM mRNA levels in stable transfectants expressing exogenous EpCAM from the CMV promoter were reduced by 43% (Supplementary 6c), suggesting a combination of transcriptional and post-translational regulation of EpCAM expression. Ectopic EpCAM over-expression had no measurable impact, neither on expression of Oct3/4, Sox2 and Nanog in pluripotent and differentiated E14TG2α cells (Fig. 5e), nor on morphology and generation rate of EBs (Supplementary Figure 6d,e). Assessment of selected ecto-, meso- and endodermal markers under pluripotency and at intermediate (d8) and late time points (d21) of spontaneous differentiation disclosed significant induction of hepatocytic markers alpha-fetoprotein (Afp) and fibronectin 1 (Fn1) in EpCAM over-expressing ESC (Fig. 5f).

Next, we addressed the influence of a loss-of-function of EpCAM on pluripotency. CRISPR-Cas9-mediated knockouts of *EPCAM* were generated as E14TG2α single cell clones and were confirmed through genomic DNA sequencing, protein and cell surface expression. Premature stop codons resulted in theoretical proteins with predicted compositions of 28 to 144 N-terminal amino acids (Supplementary Figure 7a), which led to a complete loss of EpCAM at the cell surface and in lysates (Fig. 5g). EpCAM knockout (n = 6 independent clones) resulted in reduced expression of pluripotency genes Oct3/4, Sox2 and Nanog, ranging from 20%–48%, 30%–63%, and 57%–75% reduction, respectively (Fig. 5h). Effects of EpCAM knockout on the capacity of ESC to differentiate into endodermal tissue was analyzed following bFGF and RA treatment through the assessment of endodermal markers Foxa2, Afp and Eomes. All knockout clones displayed significant >50% reductions of endodermal markers, except for clone #58, which expressed Afp similarly and Foxa2 to enhanced levels compared to wild-type E14TG2α ESC (Fig. 5i). Hence, EpCAM expression is maintained during endodermal differentiation, while EpCAM knockout reduces pluripotency and endodermal differentiation capacity.

### EpCAM regulation is mandatory for mesodermal differentiation of ESC to cardiomyocytes

**Figure 6:**
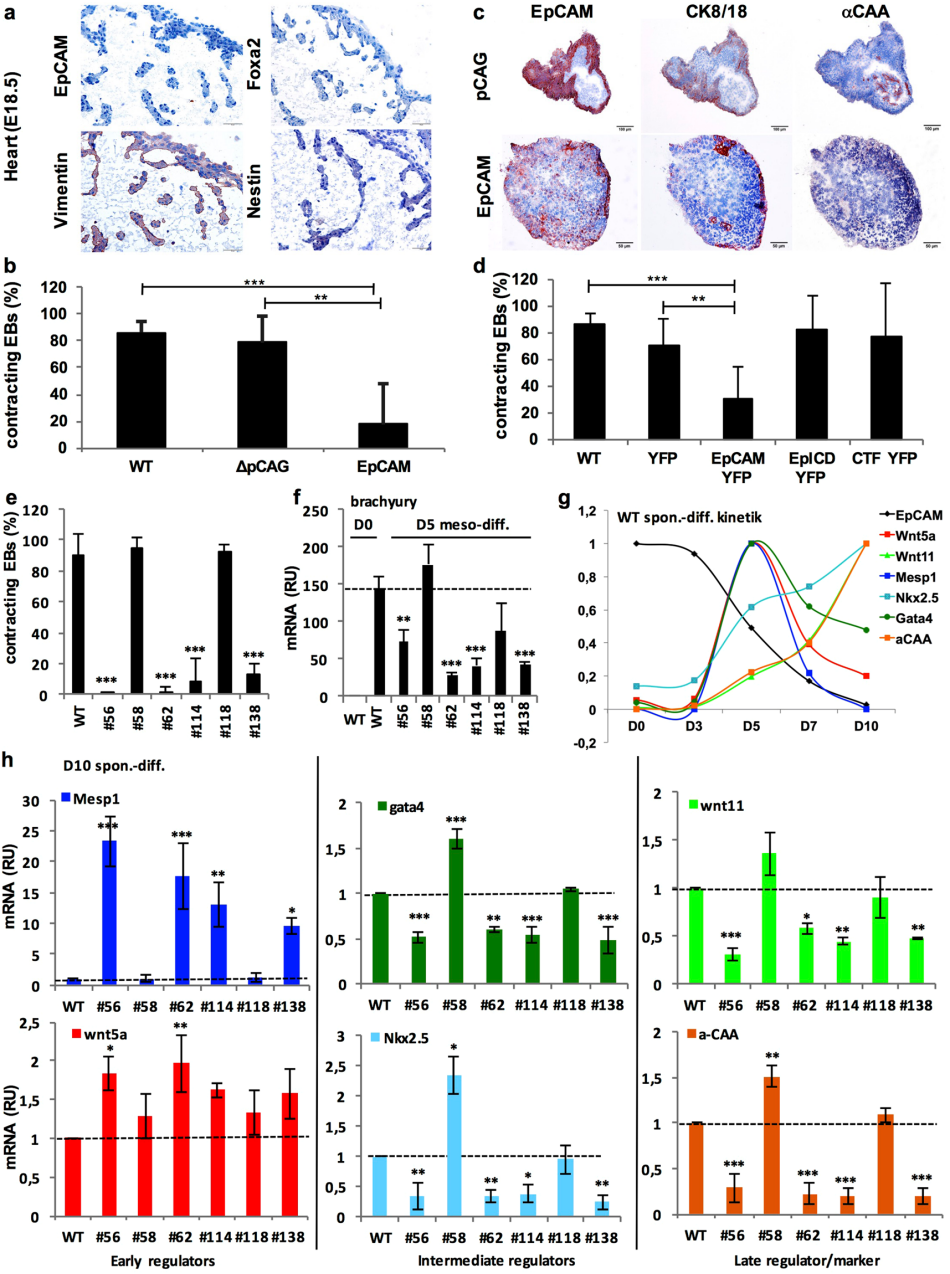
EpCAM expression regulates cardiomyocyte formation. **(a**) EpCAM, Foxa2, vimentin and nestin expression was assessed by immunohistochemistry staining in E18.5 heart of C57BL-6 mice. **(b)** Shown are percent of contracting EB of wild-type E14TG2α ESC (WT), pCAG and EpCAM transfectants at day 10 of spontaneous differentiation (n ≥ 4 independent experiments). **(c)** EpCAM, CK8/CK18 and α-CAA expression was assessed by immunohistochemistry staining in consecutive sections of contracting EB from pCAG and non-contracting EB from EpCAM-expressing E14TG2 ESC (D10). (Representative pictures of n = 3 independent experiments). Scale bars represent 100 µm (upper panels) and 50 µm (lower panels). **(d)** Percent contracting EB of wild-type E14TG2α ESC (WT), YFP, EpCAM-YFP, EpICD-YFP and CTF-YFP expressing transfectants at day 10 of spontaneous differentiation (n ≥ 4 independent experiments). **(e)** Shown are percent contracting EB of wild-type E14TG2α ESC (WT) and *EPCAM* knockout clones at day 10 of spontaneous differentiation (n ≥ 4 independent experiments). **(f)** Brachyury mRNA expression in wild-type and *EpCAM* knockout E14TG2α ESC clones was measured by quantitative PCR after mesodermal differentiation upon treatment with CHIR 99021 and cyclopamine at day 5 (n = 3 independent experiments). (**g**) Wnt5a, Mesp1, Wnt11, Gata4, Nkx2.5 and α-CAA mRNA expression was measured by quantitative PCR in wild-type E14TG2α ESC after spontaneous differentiation in a kinetic at the indicated time points (n = 3 independent experiments). **(h)** Wnt5a, Mesp1, Wnt11, Gata4, Nkx2.5 and α-CAA mRNA expression measured by quantitative PCR in wild-type and *EpCAM* knockout E14TG2α ESC clones after spontaneous differentiation (D10) (n = 3 independent experiments). Markers in (g–h) are all color-coded. Mean ± SEM; Student’s T-test (n = 2 groups) or One-Way ANOVA (n ≥ 3 groups); p < 0.05, **p < 0.01, ***p < 0.001.

*In vivo*, EpCAM was strictly lacking in nascent mesodermal progenitors (see Fig. 1) including the cardiac mesoderm (see Fig. 2). Analysis of perinatal E18.5 cardiomyocytes substantiated a lack of EpCAM, Foxa2 and nestin expression, but expression of vimentin (Fig. 6a). Frequent spontaneous 3D-differentiation of ESC to contractile cardiomyocytes^37^ was confirmed within E14TG2α EB (Video 1).

EpCAM expression in EBs closely mimicked the strict regulation observed *in vivo*, with a pattern mutually exclusive to vimentin and a lack of EpCAM in cardiomyocytes. Therefore, we sought to analyze the effects of exogeneous over-expression of EpCAM on mesodermal differentiation to cardiomyocytes. Ectopic over-expression of EpCAM resulted in reduction of the frequency of contracting EB, as a functional surrogate of cardiomyocyte differentiation comparable to foetal cardiomyocyte development^38^, from an average 86.5% in wild-type and 79% in vector controls to 17.6% in EpCAM over-expressing ESC (Fig. 6b). Immunohistochemical staining of contracting EB with antibodies specific for EpCAM, epithelial marker CK8/18 and cardiomyocyte marker alpha-cardiac actin (α-CAA) corroborated the close proximity but segregation of EpCAM^+^/CK8/18^+^ epithelial cells and EpCAM^−^/α-CAA^+^ cardiomyocytes (Fig. 6c). In non-contracting EB from EpCAM transfectants, EpCAM was expressed more evenly and overlapped with CK8/18 in marginal cells, whereas α-CAA was lacking (Fig. 6c). Conversely, inhibition of cardiomyocyte formation upon treatment with high RA concentrations (10^−7^ M) as reported in^39^ resulted in maintenance of EpCAM expression (Supplementary Figure 8). Furthermore, cardiomyocyte inhibition required full-length, uncleaved EpCAM, since only EpCAM-YFP but none of the RIP products of EpCAM cleavage, *i.e*. EpICD-YFP and EpCAM-CTF-YFP fusion proteins, inhibited the formation of contractile EB (Fig. 6d).

Thus, EpCAM over-expression inhibits cardiomyocyte formation, but RIP of EpCAM is not the molecular basis for the inhibitory effect.

### EpCAM knockout impacts on cardiomyocyte differentiation

Complete loss of EpCAM in mesodermal progenitors is required for cardiomyocyte development, but EpCAM simultaneously impacts on pluripotency and endodermal differentiation. Owing to the interdependency of cells during differentiation, we analyzed possible effects of EpCAM knockout on EB contraction. Three control single cell clones that have undergone CRISPR-Cas 9 transfection and selection procedures, but which displayed wild-type (n = 2) or only minor decrease in levels of EpCAM (n = 1) expressed levels of pluripotency genes Oct3/4 and Nanog comparable to wild-type cells, thus suggesting full pluripotency (Supplementary 6b–e). None of the control clones were impaired in cardiomyocyte formation as measured through the generation of contracting EBs (Supplementary Figure 6f). In contrast, four out of six E14TG2α EpCAM knockout clones were severely impaired in the formation of contracting EB, with contraction rates dropping to 0.1–12.5% (Fig. 6e). Guided mesodermal differentiation of these four knockout clones after treatment of cells with 30 µM CHIR 99021 and 5 µM cyclopamine for 5 days was associated with reduced levels of brachyury, demonstrating diminished capacity to form mesodermal structures (Fig. 6f).

Physical contact with endodermal cells is reportedly decisive during the generation of cardiomyocytes. Initially, mesodermal progenitors require a Mesp1/Wnt5a-dependent activation of cardiovascular development, which is followed by mandatory reduction of Wnt5a and Mesp1 expression, and subsequent induction of Wnt11 for the completion of cardiomyocyte maturation through the instruction by Sox17^+^/EpCAM^+^ endodermal cells^40–42^. Spontaneous differentiation of wild-type E14TG2α cells was conducted in a time kinetic over ten days and mRNA expression of EpCAM, Wnt5a, Mesp1 (both early regulators), Gata4, Nkx2.5 (both intermediate regulators), Wnt11 (late regulator) and α-CAA (cardiomyocyte marker) was assessed. Time-dependent expression of these genes confirmed the abovementioned sequence of expression, with a peak of Wnt5a, Mesp1 and Gata4 at day 5 of differentiation, followed by a strong or complete loss of expression of Wnt5a and Mesp-1 at day 7, respectively. Expression of Gata4 was decrease to approx. 50% at day 10. Starting with day 5, Wnt11, Nkx2.5 and α-CAA expression was sustainably increased and peaked at day 10 (Fig. 6g). After spontaneous differentiation at day 10, non-contracting knockout clones were characterized by marginally elevated levels of Wnt5a, strongly up-regulated Mesp1 expression and significantly reduced levels of Wnt11, Gata4, Nkx2.5 and α-CAA (Fig. 6h**)**.

Wnt5a and Mesp1 regulation was further substantiated by their exclusive expression in cells of the primitive streak during early gastrulation at day E6.5 (Supplementary Figure 9). Both regulators of initial cardiomyocyte development were further expressed in nascent mesoderm progenitors at day E7.0, but especially Mesp1 was strongly down-regulated in all Flk1^+^ mesodermal progeny along differentiation (Supplementary Figure 9). A role for EpCAM^+^/Gata4^+^ endodermal cells in cardiomyocyte development was further suggested by their co-expression in primitive and visceral endoderm at early stages of gastrulation (Supplementary Figure 9).

Thus, impaired regulation of EpCAM through genetic knockout impacts on endo- and mesodermal differentiation, which are both required for the formation of contracting cardiomyocytes. Eventually, EpCAM-deficient ESC only partially progressed through mesodermal differentiation, and appeared blocked at a Mesp1^high^ stage.

### EpCAM regulates ESC differentiation via ERas/AKT

**Figure 7:**
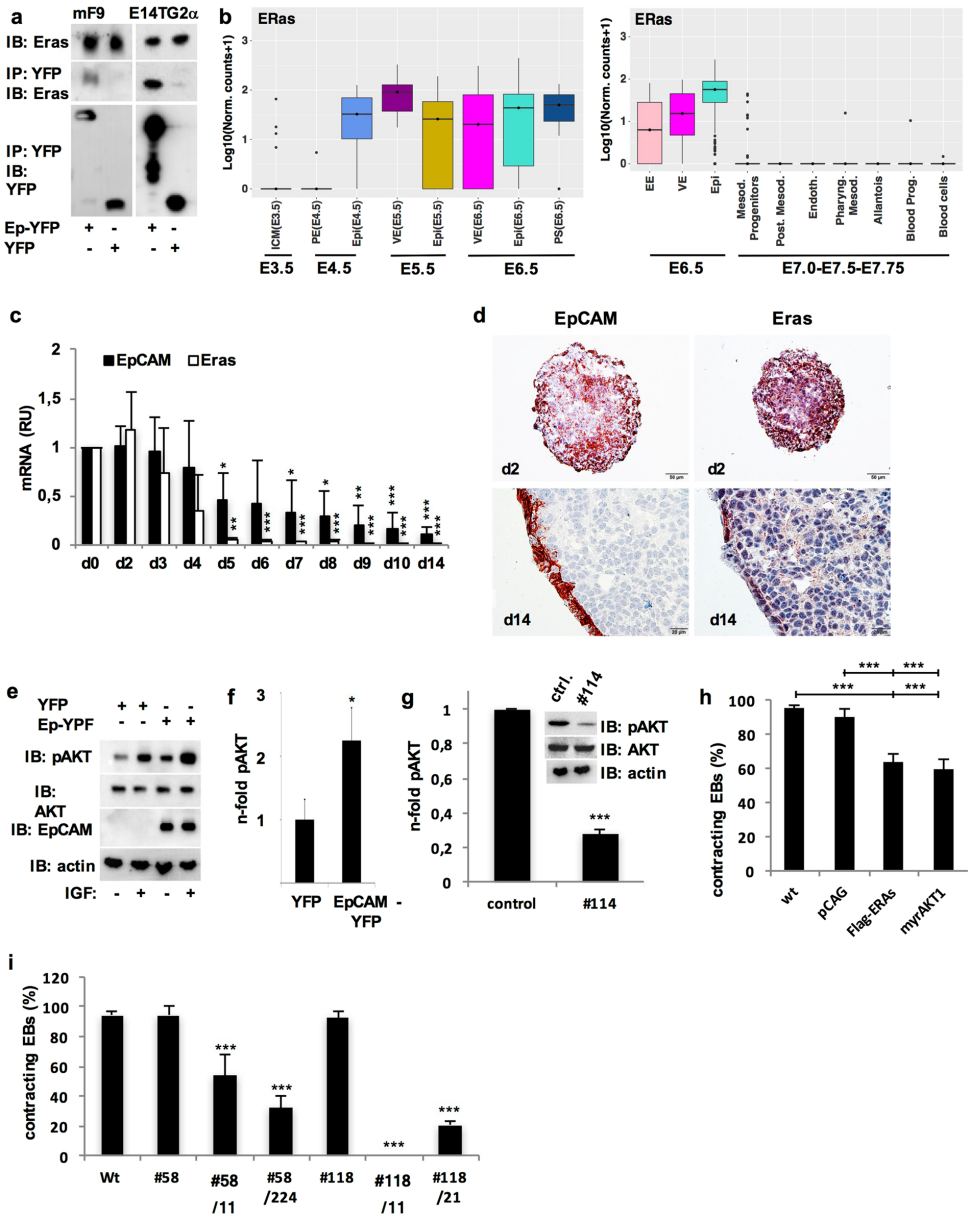
EpCAM/ERas/AKT axis limits cardiomyocyte formation. (**a**) EpCAM-YFP and endogenous ERas interact in F9 teratoma cells and E14TG2α ESC transfectants expressing EpCAM-YFP or YFP. EpCAM-YFP and YFP were immunoprecipitated from stable F9 and E14TG2α ESC transfectants. Co-precipitated endogeneous ERas was detected with in immunoblots with specific antibodies. Immunoprecipitation (IP); immunoblot (IB) (n = 3 independent experiments). (**b**) Transcript levels of ERas were assessed from RNA-sequencing datasets generated from single cells at day E3.5–6.5^32^ (left) and E6.5–7.75^33^ (right). Transcript levels are depicted as box-whisker plots with log_10_ normalised counts (adding a pseudocount of 1). See Fig. 1 and Methods for the approximate localization of cell types and for additional details on the datasets. (**c**) EpCAM and ERas mRNA expression was measured by quantitative PCR in pluripotent E14TG2 ESCs (d0) and EB (d2–14) (n = 3 independent experiments). (**d**) EpCAM and ERas protein expression was assessed by immunohistochemistry in E14TG2α ESC embryoid bodies (EB d2, d14) (n = 3 independent experiments). Scale bars represent 50 µm (d2) and 20 µm (d14). (**e**) AKT phosphorylation was assessed by immunobloting with the indicated antibodies in E14TG2α ESC expressing EpCAM-YFP or YFP, with or without treatment with insulin-like growth factor (IGF) (n = 3 independent experiments). (**f**) Quantification of AKT phosphorylation. Shown are mean n-fold changes normalized for AKT phosphorylation levels in control YFP transfectant cells (n = 3 independent experiments). (**g**) AKT phosphorylation was assessed by immunobloting with the indicated antibodies in wild-type E14TG2α ESC and knockout clone #114. Graph represents the quantification of AKT phosphorylation in wild-type E14TG2α ESC and knockout clone #114 (n = 3 independent experiments). **(h)** Shownare mean percent contracting EB of wild-type E14TG2α ESC (WT), pCAG, Flag-ERas and myrAKT transfectants (n = 6 independent experiments). **(i)** Shown are mean percent contracting EB of wild-type E14TG2α ESC (WT), single EpCAM (#58, #118) and double EpCAM/ERas (#58/11, #58/224, #188/11, #118/21) knockout clones (n = 3 independent experiments). Mean ± SEM; Student’s T-test (n = 2 groups) or One-Way ANOVA (n ≥ 3 groups); p < 0.05, **p < 0.01, ***p<0.001.

EpCAM cleavage products CTF and EpICD did not limit cardiomyocyte formation, suggesting a role for full-length EpCAM in inhibition. Interacting partners of full-length EpCAM were assessed using a combination of stable isotope labeling with amino acids in cell culture (SILAC), immunoprecipitation of YFP- and EpCAM-YFP in murine F9 teratocarcinoma cells, and identification of co-precipitated proteins by LC-MS/MS. ESC-expressed Ras (ERas), a hyperactive version of the small GTPase Ras^43^, was reproducibly identified through immunoprecipitation with EpCAM and subsequent mass spectrometry analyses as interaction partner in three independent experiments. EpCAM and ERas interaction was subsequently validated in independent co-immunoprecipitations of lysates from YFP- and EpCAM-YFP-expressing F9 teratoma cells and E14TG2α ESC (Fig. 7a). Similarly to EpCAM, single cell RNA-sequencing analysis revealed that ERas expression was lost in nascent mesodermal progenitors and later stages of mesodermal differentiation (Fig. 7b). Down-regulation of ERas was confirmed during 3D-differentiation of E14TG2α ESC (Fig. 7c) and expression was predominant in marginal cells of EB, whereas cells of inner areas were deprived of ERas at later differentiation stages (Fig. 7d). *In vivo*, EpCAM and ERas displayed a high degree of co-regulation that resulted in the simultaneous expression in kidney, hair follicles and lung, but complete loss in brain, heart and bones at day E18.5 of embryonic development (Supplementary Figure 10).

ERas signaling primarily induces the PI3-kinase/AKT branch^43^. Accordingly, overexpression of EpCAM in E14TG2α ESC induced an average 2.2-fold increase in AKT phosphorylation at serine^473^ and hyper-activated AKT after insulin-like growth factor treatment (Fig. 7e,f). Oppositely, knockout of EpCAM in ESC resulted in reduced phosphorylation of AKT by 72.5% in average (Fig. 7g). Next, FLAG-tagged ERas and a constitutively active, myristoylated variant of AKT (myrAKT) were expressed in ESC (Supplementary Figure 7g,h). Both, Flag-ERas and myrAKT significantly reduced percentages of contraction by 30% compared to wild-type and vector controls (Fig. 7h).

In order to assess whether hyperactive ERas could complement for the differentiation defects observed upon loss-of function of EpCAM, EpCAM knockout clones #58 and #118, which displayed a retained contraction capacity, were subjected to CRISPR-Cas9-mediated knockout of ERas. Two EpCAM^−^/ERas^−^ knockout clones of clones #58 and #118 were further analyzed (n = 4). Genomic DNA analysis proved genetic deletions in the ERAS locus, and double-knockout clones lacked ERas protein (Supplementary Figure 7i,j). Double knockout of EpCAM and ERas resulted in complete or substantial impairment to form contracting EB (0–54%) as compared with single-knockout clones #58 and #118clones (94% and 93%) (Fig. 7i). Impaired cardiomyocyte formation in double-knockout clones was further accompanied by reduced Gata4 expression (data not shown). Hence, EpCAM/ERas/AKT compose a regulatory signaling axis in ESC and ERas can partially complement for EpCAM knockout during differentiation.

## Discussion

In the present study, we have comprehensively addressed timing and rationale for the strict differential regulation of EpCAM throughout development. RNA-seq. datasets^32,33^, which we have re-analyzed, firstly provided a high resolution of the precise timing of EpCAM regulation from early- to mid-gastrulation embryos at the single cell level. Throughout blastocyst and initiating gastrulation stages (E3.5–6.5), EpCAM was retained at high levels in cells of the inner cell mass, primitive and visceral endoderm, epiblast, and primitive streak. Co-expression of EpCAM with endodermal transcription factor Foxa2 was observed in primitive and visceral endoderm, as well as in the primitive streak, although to lower degree. This co-expression was further substantiated in visceral endoderm and endodermal clusters in later stage embryos and in ESC-derived EB at early time points of 3D-differentiation, when formation of visceral endoderm was reported^44^. High expression of EpCAM was retained in epiblast, visceral endoderm and extraembryonic ectoderm at E6.5. At this time point, co-expression of EpCAM with mesodermal marker vimentin was mostly restricted to the primitive streak, where significant but low levels of vimentin expression emerged within the cells that will ultimately undergo gastrulation and generate all three germ layers. Genetic knockout of murine *EPCAM* in ESC performed in the present study demonstrated that expression in ESC is required for full pluripotency to form cells of all germ layers, which is in accordance with its reported role in human and murine ESC and porcine iPS^3–5,21^. Knockout clones further displayed reduced capacity for guided endodermal and mesodermal differentiation, suggesting a requirement for EpCAM function(s) for the generation of primitive and visceral endoderm, and support of mesodermal lineages.

Noteworthy, extensive loss of EpCAM expression was observed in nascent mesodermal progenitors at E7.0. Later stages of mesodermal differentiation were all characterized by complete loss of EpCAM and progressive increase of vimentin. Complete loss of EpCAM was observed in posterior mesoderm cells, demonstrating a very strict and timely down-regulation in earliest Flk1^+^ mesodermal lineages as well as later, blood progenitors and embryonic blood cells. Continuation of a strict regulation of EpCAM was verified in mid-gestation and perinatal embryos. Retention in endodermal tissue, but complete loss of EpCAM in meso- and ectodermal tissues was consistent, resulting in segregation of EpCAM^+^ and EpCAM^−^ cell clusters at E9.5, E12.5 and E18.5. Thus, repression of EpCAM in mesodermal progenitors and retention in endodermal progenitors represents an early event during gastrulation of mouse embryos.

The rationale for this repression of EpCAM in differentiation was thereafter analyzed in a 3D-differentiation model of murine ESC, which mimics aspects of early embryogenesis and allows for genetic manipulations^45^. ESC-derived EB represent a valuable approximation of an embryo-like architecture composed of an external primitive/visceral endoderm and internal meso- and ectoderm lineages^34,46^, which includes the formation of an anteroposterior axis and a primitive streak based on Wnt signaling^47,48^. Spatiotemporal patterning, with segregation but close proximity of EpCAM^+^ and EpCAM^−^ clusters, was reproduced in EB. Segregation of EpCAM^+^ and EpCAM^−^ clusters might result from a direct effect of EpCAM on cell adhesion^49^ or *via* its negative impact on E-cadherin-mediated cell-cell contacts^50^, which is a central cell adhesion molecule during asymmetric segregation of murine epithelial cells^51^ and during gastrulation of zebrafish through a Wnt11-dependent regulation of cohesion of early meso-endoderm^52^.

**Figure 8:**
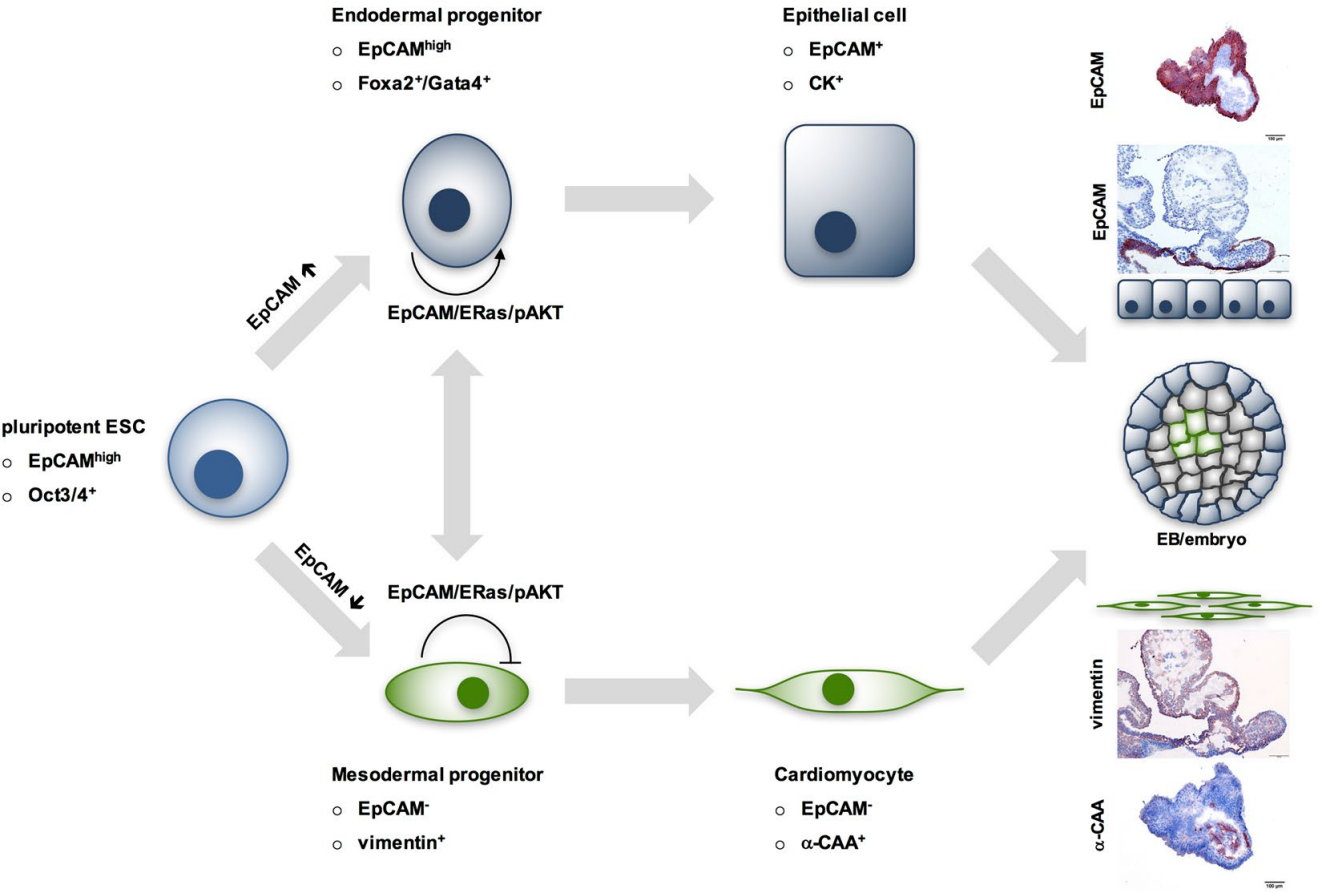
Schematic illustration of EpCAM expression in differentiating ESC.

Gain- and loss-of-function manipulations in the present study disclosed that selective expression of EpCAM supports an interdependent differentiation along EpCAM^+^ endodermal and EpCAM^−^ mesodermal lineages. In the frame of a guided endodermal differentiation protocol of ESC and during spontaneous formation of endodermal clusters, EpCAM was maintained, whereas complete repression of EpCAM was necessary in the mesodermal lineage in order to differentiate to cardiomyocytes (Fig. 8). EpCAM expression was supportive of the complete expression of Gata4, Foxa2 and Afp in ESC-derived endodermal cells. This finding is congruent with a central role of Gata4 in hepatocyte induction^53^, and a necessary physical contact of developing cardiomyocytes with endodermal cells^54^, more precisely with Gata4-producing Sox17^+^-EpCAM^+^ visceral endoderm or hepatocyte-like oval cells, to instruct cardiomyocyte differentiation^40,55^. Sox17^+^-EpCAM^+^ visceral endodermal cells themselves progress to hepatocyte progenitors^40^, further substantiating an interdependence of EpCAM^+^ and EpCAM^−^ cells in differentiation. Accordingly, high expression of EpCAM is a feature of human hepatocytic stem cells^56,57^ and EpCAM is a de-repressor of the Wnt signaling cascade that is required to license hepatic development in zebrafish^58^. Knockout of EpCAM in zebrafish induces defective liver development^58^, whereas forced expression of EpCAM in ESC fostered transcription of hepatocytic markers Afp and Fn1, as shown here.

Transcription factor Mesp1 is central to human cardiovascular development^42^ and is required for cardiomyocyte development through the regulation of cardiac mesoderm at E6.5, leading to the formation of the first heart field and, subsequently, the heart tube^42,59,60^. In the process of migration to form the heart crescent, cardiomyocyte progenitors will down-regulate Mesp-1 to further promote the generation of mature cardiomyocytes^61^. EpCAM knockout clones displayed strongly enhanced levels of Mesp1 and significantly reduced levels of Wnt11 at day 10, a time point when cardiomyocyte differentiation is completed in EB and Mesp1 down-regulation is accomplished. From these knockout studies, we conclude that EpCAM-deficient clones are blocked at a Mesp1^+^ stage along the differentiation into cardiomyocytes. The resulting reduction of Wnt11 levels will potentially impact on meso-endoderm cohesion^52^, and could ultimately impede on mesodermal differentiation.

Promoting and restricting effects of EpCAM on differentiation were connected to its interaction with hyperactive Ras GTPase ERas, which is central to oncogenic and proliferative features of ESC^43,62^. In line with the ability of ERas to induce activation of AKT, expression of EpCAM fostered the activating phosphorylation of AKT at serine^473^. Over-expression of ERas or an activated version of AKT mimicked limiting effects of EpCAM on cardiomyocyte formation, although less potently than EpCAM. This is congruent with a reported loss of ERas in E7.5 embryos to facilitate primitive streak and mesoderm generation, but retention in endoderm of the same gestation stage^63^. Regulation of ERas was even stricter than that of EpCAM, with a total loss already at the stage of nascent mesodermal progenitors. EpCAM knockout clones with retained capability of cardiomyogenesis were severely impaired after additional ERas knockout. This suggests that ERas can complement for the loss of EpCAM to support the formation of Gata4^+^ endodermal cells, which are required for cell non-autonomous inductive signals to cardiomyocyte progenitors (Fig. 8). EpCAM is together with galectin-1 the second membrane receptor described to interact with ERas^62^. Binding of ERas to EpCAM might facilitate initiation of signaling and recruitment of downstream molecules to ERas and, thereby, orchestrate signalling to AKT and further downstream targets. Similar roles of EpCAM and ERas are further substantiated through comparable effects during somatic reprogramming of fibroblasts to induced pluripotent stem cells^20,22,64^. Thus, EpCAM/ERas/AKT represents a novel signaling axis in ESC that participates in the regulation of endodermal cells and limits mesodermal differentiation to cardiomyocytes.

In summary, EpCAM expression is tightly regulated at earliest time points of gastrulation in order to achieve a mandatory spatiotemporal cellular heterogeneity of EpCAM in endo- and mesodermal lineages. Cell-autonomous roles of EpCAM emerge as a licensing factor for endoderm and as a limiting factor of cardiomyocyte development. Simultaneously, cell non-autonomous functions of EpCAM are likely in effect, such that EpCAM^−^ mesodermal cells depend on EpCAM^+^ Gata4-producing cells during the regulatory interplay of cardiomyocyte development (Fig. 8). Disturbance of this tight control of EpCAM results in perturbation of spontaneous, endo- and mesodermal differentiation.

## Methods

### Biological and technical replicates

Throughout the manuscript, biological replicate is referred to as a fully independent experiment performed with newly generated material (*e.g*. cell lysates, mRNA, etc.), whereas a technical replicate (*e.g*. mentioned as “duplicates”) is a repeated measurement with identical material. Technical repeats address assay accuracy and reproducibility (assay noise), while biological repeats demonstrate reproducibility of assay outcome/results^65^.

### RNA-sequencing dataset analysis

The two single-cell RNA-sequencing datasets we used, were previously published in^32,33^. The first dataset (Fig. 1a) includes cells from mouse embryos at the following stages: E3.5 (n = 10 embryos, n = 99 cells), E4.5 (n = 5 embryos, n = 105 cells), E5.5 (n = 9 embryos, n = 267 cells), E6.5 (n = 11 embryos, n = 250 cells)^32^. The second dataset (Fig. 1b) includes 1,205 single cells, collected from the epiblast at E6.5 and from E7.0, E7.5, E7.75 stages; cells at E7.0 were sorted for Flk1, which marks much of the developing mesoderm, whereas at E7.5 and E7.75 Flk1^+^, Flk1^+^/CD41^+^ and Flk1^−^/CD41^+^ cells were collected (CD41 is up-regulated during blood development)^33^. Each dataset was normalized for sequencing depth by using DESeq size factors^66^. To visualize the data, we used t-stochastic neighbor embedding dimensionality reduction^67^ (“Rtsne” function from the R package “RtSNE” version 0.13, default options^68^) on the dissimilarity matrix, defined as (1 − ρ)/2, ρ being the Spearman correlation coefficient between pairs of cells. This correlation coefficient was calculated by using only the highly variable genes in each dataset, which we identified by applying the method described in^69^. The clusters highlighted in Fig. 1 are those that were reported in the original publications including the data^32,33^. Transcript levels were depicted as box-whisker plots with log10 normalized counts (adding a pseudo-count of 1).

### Cell lines, CRISPR-Cas9 manipulation

E14TG2α and murine teratoma F9 cells were cultured and stably transfected as described before^19^. E14TG2α and Bruce4 cells were cultured in Stempan Gmem medium (PAN-Biotech, Aidenbach, Germany) and leukemia inhibitory factor (ESGRO®LIF; 60,000 U/mL; Becton Dickinson, Heidelberg, Germany) on 0.1% gelatin-coated 6-well plates. Murine F9 teratoma cells were cultured in Dulbecco’s modified Eagle’s medium (DMEM, high glucose) supplemented with 20% FCS (Biochrom AG, Heidelberg, Germany) and 1% penicillin/streptomycin.

All cDNAs were PCR-amplified and cloned in 141pCAG-3SIP expression vector using Nhe1/EcoR1 restriction sites. Myristylated Akt was a kind gift from Prof. D. Saur (TUM, Munich, Germany).

Knockout of EpCAM and ERas was conducted using the CRISPR-Cas9-based system (Sigma Aldrich, Munich, Germany). Two guide RNAs (gRNAs) located in exons 2 and 4 of the *EPCAM* gene and two gRNAs located in exon 1 of ERAS were used in the all-in-one Cas9 and guide RNA expression plasmids. After transfection, plasmid-positive E14TG2α cells were sorted according to GFP expression and deposited as single cells into 96-well plates. Single-cell clones were analyzed through flow cytometry and immunobloting. Genetic knockout was confirmed upon sequencing of the according genomic DNA flanking and encompassing gRNA sequences.

### Embryoid body formation and contraction

To generate EB, 500 cells in 20 µl of Stempan Gmem medium (PAN-Biotech, Aidenbach, Germany) lacking ESGRO®LIF were plated on the lid of a cell culture plate according to^46^. After three days, EB were manually transferred to ultra-low attachment plates (Nunc, Wiesbaden, Germany) for four days before transfer in standard 96 well plates for further differentiation up to 21 days. For immunohistochemistry, EB were embedded in tissue-tek (Sakura Finetek, Germany), snap-frozen in liquid nitrogen and processed to 4 µm sections. EB contraction was analyzed after 10 days *via* counting under a microscope in 96-well plates. At this time point, EB had an average size of 466 ± 24 µm (mean ± SEM; of ≥3 independent experiments). Contraction is given as percentage of contracting EBs standardized for number of EBs formed.

### Embryo isolation

Embryos were isolated from the uterus of C57BL/6 wild-type mice at days *post coitum* E9.5, E12.5, and E18.5. All methods involving animals were carried out in accordance with relevant guidelines and regulations of the animal care licensing committee of the Helmholtz Centre Munich. All experimental protocols were approved by the licensing committee of the Helmholtz Centre Munich and the state administration “Regierung von Oberbayern”.

### Mesodermal and endodermal differentiation

Mesodermal differentiation was induced *via* a modified protocol from Kanke *et al*.^70^. ESC (100.000 cells/well) were seeded in 6 well plates in Stempan E14 GMEM medium (Roche, Mannheim, Germany) containing ESGRO®LIF (60,000 U/mL; Becton Dickinson, Heidelberg, Germany), 37 °C, 5%CO_2_, before starting differentiation. After 24 hrs, cells were thoroughly washed with PBS and differentiated in medium w/o LIF supplemented with 30 µM CHIR 99021 (Sigma, St.Louis, MO, USA) and 5 µM Cyclopamine (Selleckchem.com, Houston, TX, USA). After Incubation for 5 days at 37 °C, 5%CO_2_ cells were trypsinized and harvested.

Endodermal differentiation was induced according to^35^. ESC (13.000 cells/cm^2^) were cultured in Stempan E14 GMEM medium supplemented with 10^−7^ M all-trans retinoic acid (Sigma, St.Louis, MO, USA) and 25 ng/mL bFGF (Thermo Fisher, Waltham, MA, USA) for 3 days at 37 °C, 5%CO_2_. At 70–80% confluence, cells were diluted 1:10 and cultured in medium supplemented with 25ng/ml bFGF at 37 °C, 5%CO_2_. After a total of 12 days, cells were trypsinized and harvested for RT-PCR.

### Flow cytometry, immunohistochemistry and immunoblot staining

Cell surface expression of EpCAM and SSEA1 was measured as described^5^. Briefly, cells were stained with the EpCAM-specific antibody (CD326; BD Biosciences; 1:50 dilution in PBS-3% FCS) or the stage-specific mouse embryonic antigen (SSEA)-1-specific antibody (mouse polyclonal antibody MC480; Abcam) for 15 minutes on ice, washed three times in PBS-3% FCS, and stained with fluorescein isothiocyanate-conjugated rabbit anti-mouse secondary antibody (Vector Laboratories; FI-4001). Measurement of cell surface expression of EpCAM was performed in a FACSCalibur device (BD Pharmingen, Heidelberg, Germany).

Embryos from C57BL-6 mice at gestation stages E9.5, E12.5 and E18.5 were embedded in tissue-tek (Sakura Finetek, Germany) and snap frozen in liquid nitrogen before sectioning to 4 µm thick sections. Immunostaining of EpCAM (Becton Dickinson, Germany; #552370), vimentin (Abcam, USA; #ab92547), Foxa2 (Novus Biologicals, Germany; #NBP1-95426), nestin (Sigma, Germany; N5413), CK8/18 (Progen Immunodiagnostic, Germany; #GP11), ERas (Santa Cruz, USA; sc-51072), α-CAA (Sigma, Germany; AC1-2042), and Gata4 (Ebioscience, USA; #14-9980) was performed using avidin-biotin-peroxidase complex method (Vectastain, Vector laboratories, Burlingame, CA, USA).

Immunobloting of EpCAM, ERas, Akt and phospho-Akt-Ser^473^ (Cell Signaling, USA; #92725 and #4060) was performed with 10–50 µg of whole cell lysate (PBS, 1% triton X-100, Roche complete protease inhibitors). Proteins were separated in a 10–15% SDS-PAGE before transfer on PVDF membranes (Millipore, Germany). Primary antibodies were detected with HRP-conjugated secondary antibodies and ECL (Merk Millipore, Darmstadt, Germany).

### SILAC screen

Murine F9 cells expressing EpCAM-YFP or YFP were cultured 14 days in Silantes medium containing heavy (lysine-8/arginine-10) and light amino acids (lysine-0/arginine-0), respectively. In three biological repeats, 3 mg (exp. #1 and #2) or 7mg (exp. #3) whole cell lysate of each cell line were incubated with 30 µL GFP-Trap® agarose beads (3 hrs, 4 °C, rotation), washed in 700 µl 0.2% tween in phosphate buffered saline. Samples from EpCAM-YFP and YFP immunoprecipitates were pooled in three independent experiments and proteins recovered upon heating at 95 °C, 5 min in Laemmli buffer. Immunoprecipitated proteins were separated on SDS-PAGE, trypsinized by in-gel digestion, and analyzed via LC-MS/MS on a LTQ Orbitrap XL coupled to an Ultimate 3000 nano-HPLC. SILAC data analysis was performed using the Max Quant software as described previously^71^. Potential interaction partners were defined as proteins enriched by ≥3-fold, p-values ≤0.05, and ≥2 unique peptides in all three independent experiments. Statistics performed was a classical two-sided unpaired t-test on the individual protein intensities per label per sample, with the intensities for replicate #3 divided by 2.33 in order to normalize for differences in protein input in the IP.

### Quantitative RT-PCR

Total mRNA was prepared using RNeasy Mini Kit (Qiagen, Hilden, Germany) and reverse transcribed with the QuantiTect Reverse Transcription-Kit (Qiagen, Hilden, Germany). cDNA was amplified using SYBR-Green PCR mastermix (Qiagen, Hilden, Germany) and gene specific primers. Normalizations across samples were performed using average of constitutive gene expression of glucuronidase beta (gusb). Gene expression levels were calculated according the equation 2-ΔΔCT, where ΔCT was defined as CT_gene_ of interest – CT_endogenous_ control. Levels of mRNA transcripts were assessed upon real time quantitative PCR with a LightCycler 480 device and LightCycler 480 SYBR Green II Master mix (Roche, Mannheim, Germany).

Afp: FW 5′-CTTCCCTCATCCTCCTGCTAC-3′; BW 5′-ACAAACTGGGTAAAGGTGATGG-3′

Brachyury: FW 5′-ACAGAGAGCGCAGGGAAGAG-3′; BW 5′-GTGGTCGTTTCTTTCTTTGGC-3′

α-CAA: FW 5′-CTGGATTCTGGCGATGGTGTA-3′; BW 5′-CGGACAATTTCACGTTCAGCA-3

EpCAM: FW 5′-CAGTGTACTTCCTATGGTACACAGAATACT-3′; BW 5′-CTAGGCATTAAGCTCTCTGTGGATCTCACC-3′

Eomes: FW 5′- GCGCATGTTTCCTTTCTTGAG-3′; BW 5′- GGTCGGCCAGAACCACTTC-3′

ERas: FW 5′- TGCCTACAAAGTCTAGCATCTTG-3′; BW 5′- CTTTTACCAACACCACTTGCAC-3′

Fibronectin: FW 5′- CCTCCTGATAGTGTGGTGTCTG-3′; BW 5′- CGTACACCCAGCTTGAAGCCAAT-3′

Foxa2: FW 5′- GGAAGGGCACGAGCCATCCG-3′; BW 5′- AGTTCTGCCAGCGCTGCTGG-3′

Gata4: FW 5′- GCCTGTATGTAATGCCTGCG-3′; BW 5′-CCGAGCAGGAATTTGAAGAGG-3′

GusB: FW 5′-CAACCTCTGGTGGCCTTACC-3′; BW 5′-GGGTGTAGTAGTCAGTCACAGAC-3′

Mesp1: FW5′- GCTCGGTCCCCGTTTAAGC-3′; BW 5′- ACGATGGGTCCCACGATTCT-3′

Nanog: FW 5′- TCTTCCTGGTCCCCACAGTTT-3′; BW 5′- GCAAGAATAGTTCTCGGGATGAA-3′

Nestin: FW 5′- GGAGAGTCGCTTAGAGGTGC-3′; BW 5′- GGTGGGGGTTCTGGCCTTAA -3′

Nkx2.5: FW 5′- GACAAAGCCGAGACGGATGG-3′; BW 5′- CTGTCGCTTGCACTTGTAGC-3′

Oct3/4: FW 5′-ATCGGACCAGGCTCAGAGGTATTG-3′; BW 5′-GTTCTCATTGTTGTCGGCTTCC-3′

Sox2: FW 5′- GACAGCTACGCGCACATGA-3′; BW 5′- GGTGCATCGGTTGCATCTG-3′

Wnt5a: FW 5′- CTCTAGCGTCCACGAACTCC-3′; BW 5′- CAAATAGGC AGCCGAGAGAC-3′

Wnt11: FW 5′- TTGACCTGGAGAGAGGTACAC-3′; BW 5′- GTCAGGGGAGCTCTGTAGATA-3′.

### Chromatin immunoprecipitation

Chromatin immunoprecipitation was performed with pluripotent ESCs and after differentiation in 3D-cultures according to a published protocol^72^. Immunoprecipitation was conducted with polymerase 2 (Santa Cruz, Santa Cruz, USA; sc-899), H3K4me3 and H3K27me3 specific antibodies (Millipore, Germany; #17-614 and #17-622).

EpCAM-1 (ChIP): FW 5′-CGCAGCGCAAAGTCAAGTAT-3′; BW 5′- ACGTGAAAGCCGAAAGGGAT-3′

EpCAM-2 (ChIP): FW 5′- ATTGGAAAGTAAGCTGCCAGG-3′; BW 5′- GGACGAGACTGGACGTGAAA-3′

CenpI (ChIP): FW 5′- AACTCACGGATATTGAAGTGCAT-3′; BW 5′- CACAGAGCCAGGATACTGCTT-3′.

### Statistics

Results are presented as mean ± SEM of ≥3 independent experiments. Significance of differences of two groups was calculated using a Student’s T-test in Excel. Significance of differences between more than two groups were calculated with a One-Way ANOVA test and subsequent post-hoc multiple comparisons including the Bonferroni correction in SPSS. Levels of significance were displayed as *p-value < 0.05; **p-value < 0.01; ***p-value < 0.001, and referred to wild-type conditions unless depicted otherwise.

## Electronic supplementary material

Supplementary Information

Video 1
