# Supplementary material for: Spatiotemporal patterning of EpCAM is important for murine embryonic endo- and mesodermal differentiation
Source: Sci Rep. 2018 Jan 29;8:1801. doi: 10.1038/s41598-018-20131-8 (PMC5789065; doi:10.1038/s41598-018-20131-8)
Supplement: Supplementary file 1 — Supplementary Information [file 41598_2018_20131_MOESM1_ESM.pdf]

Supplementary information for:

Spatiotemporal patterning of EpCAM is important for murine embryonic endo- and mesodermal differentiation

Sannia Sarrach[1,#], Yuanchi Huang[1,2,#], Sebastian Niedermeyer[1], Matthias Hachmeister[1], Laura Fischer[1], Sebastian Gille[1], Min Pan[1], Brigitte Mack[1], Gisela Kranz[1], Darko Libl[1], Juliane Merl-Pham[3], Stefanie M. Hauck[3], Elisa Paoluzzi Tomada[1], Matthias Kieslinger[4,5], Irmela Jeremias[4,6], Antonio Scialdone[7,8,9], Olivier Gires[1]

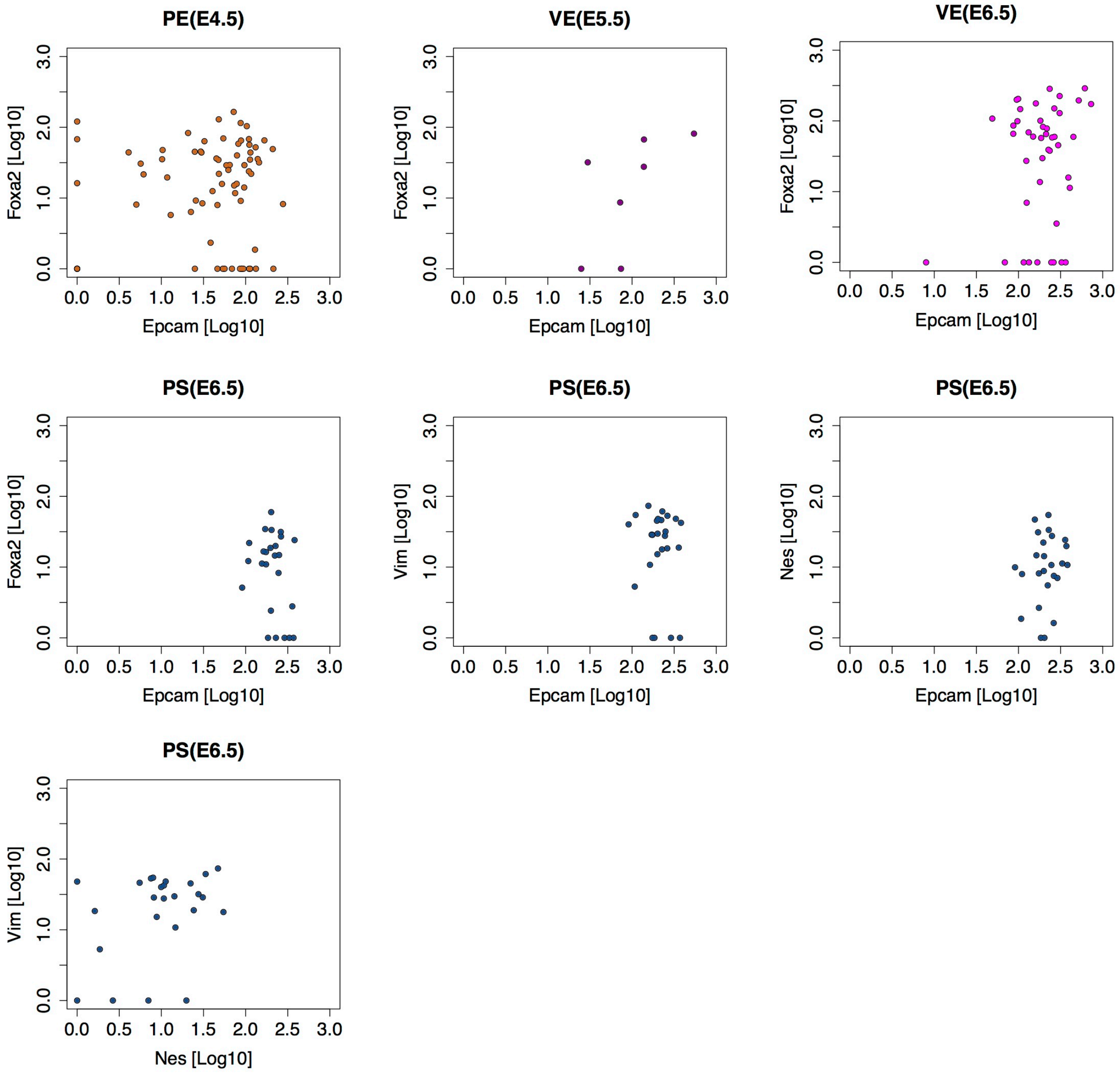

Supplementary Figure 1. Co-expression of EpCAM, Foxa2, vimentin and nestin in early gastrulation.

Co-expression patterns of the indicated transcripts are depicted as scatter plots (log10) in primitive endoderm (PE), visceral endoderm (VE), and primitive streak (PS) at the indicated stages.

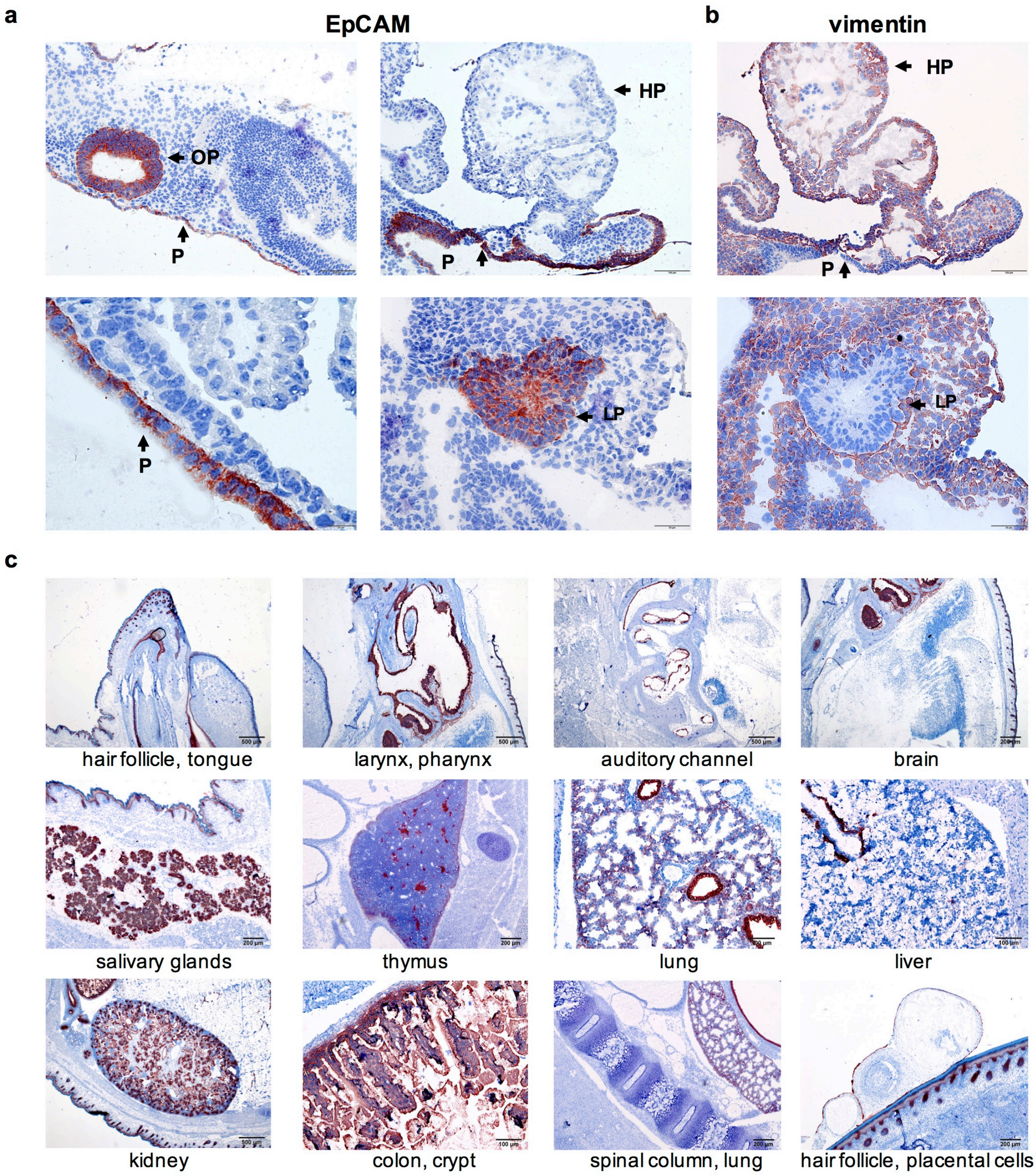

Supplementary Figure 2. EpCAM expression in mouse embryos

(**a**) EpCAM expression in C57BL-6 embryos at gestation time point E9.5 in otic pit (OP), periderm (P) and liver primordium (LP). (**b**) Vimentin expression in embryos at gestation time point E9.5 in heart primordium (HP) and liver primordium (LP). (**c**) EpCAM expression in embryos at gestation time point E18.5 in the indicated organs and structures.

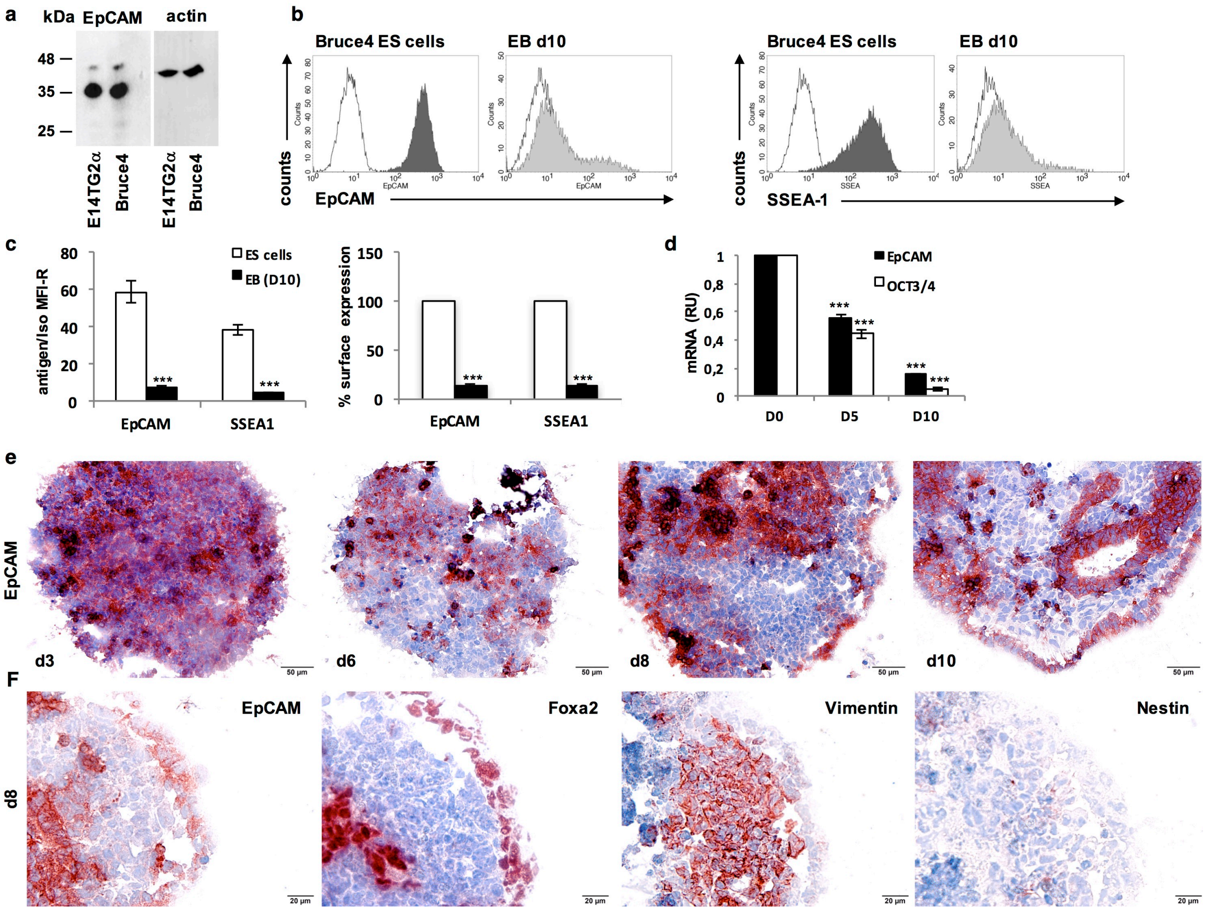

Supplementary Figure 3. EpCAM regulation in Bruce4 ESC

(**a**) EpCAM expression was assessed in whole cell lysates of E14TG2α and Bruce4 ESC. Equal loading was demonstrated upon staining of actin. (**b**) EpCAM expression was assessed on the cell surface of Bruce4 ESC upon flow cytometry using specific antibodies. Shown are representative diagrams of EpCAM expression under pluripotent conditions (ES cells) and after ten days of spontaneous differentiation in EB (EB d10). (**c**) Quantification of EpCAM and SSEA-1 expression in Bruce4 ESC under pluripotency conditions (ES cells) and after ten days of spontaneous differentiation in EB (EB d10). Shown are mean fluorescence intensity ratios (left panel) and percentages (right panel) (n=3 independent experiments). (**d**) EpCAM and Oct3/4 mRNA expression measured by quantitative PCR in pluripotent E14TG2 ESCs (d0) and EB (d5-10) (n=3 independent experiments). (**e**) EpCAM immunohistochemistry staining (brown) in three-dimensionally differentiated EB of Bruce4 ESC (d3-10). (**f**) EpCAM, Foxa2, vimentin and nestin immunohistochemistry staining (brown) in consecutive sections of of Bruce4 ESC EB (d8). Mean ±SEM; Student´s T-test (n=2 groups) or One-Way ANOVA (n≥3 groups); p<0.05, ** p<0.01, *** p<0.001.

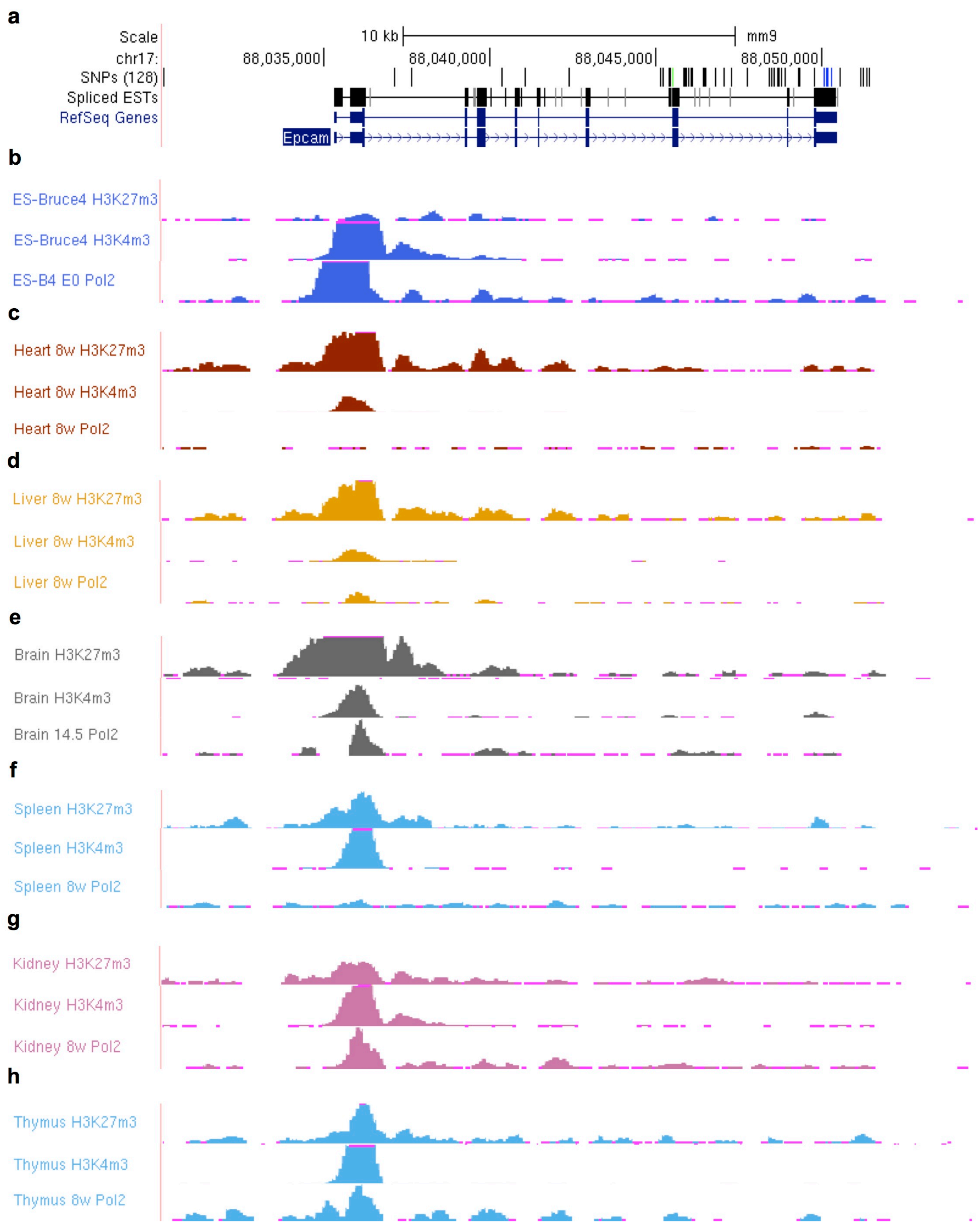

Supplementary Figure 4. Pol2 binding, H3K4 and H3K27 modifications at the EPCAM promoter

Polymerase II, H3K4 and H3K27 occupancy at the transcription start site of (**a**) the murine *EPCAM* gene in (**b**) Bruce4 embryonic stem cells, (**c**) heart, (**d**) liver, (**e**) brain, (**f**) spleen, (**g**) kidney and (**h**) thymus of 8 weeks old mice.

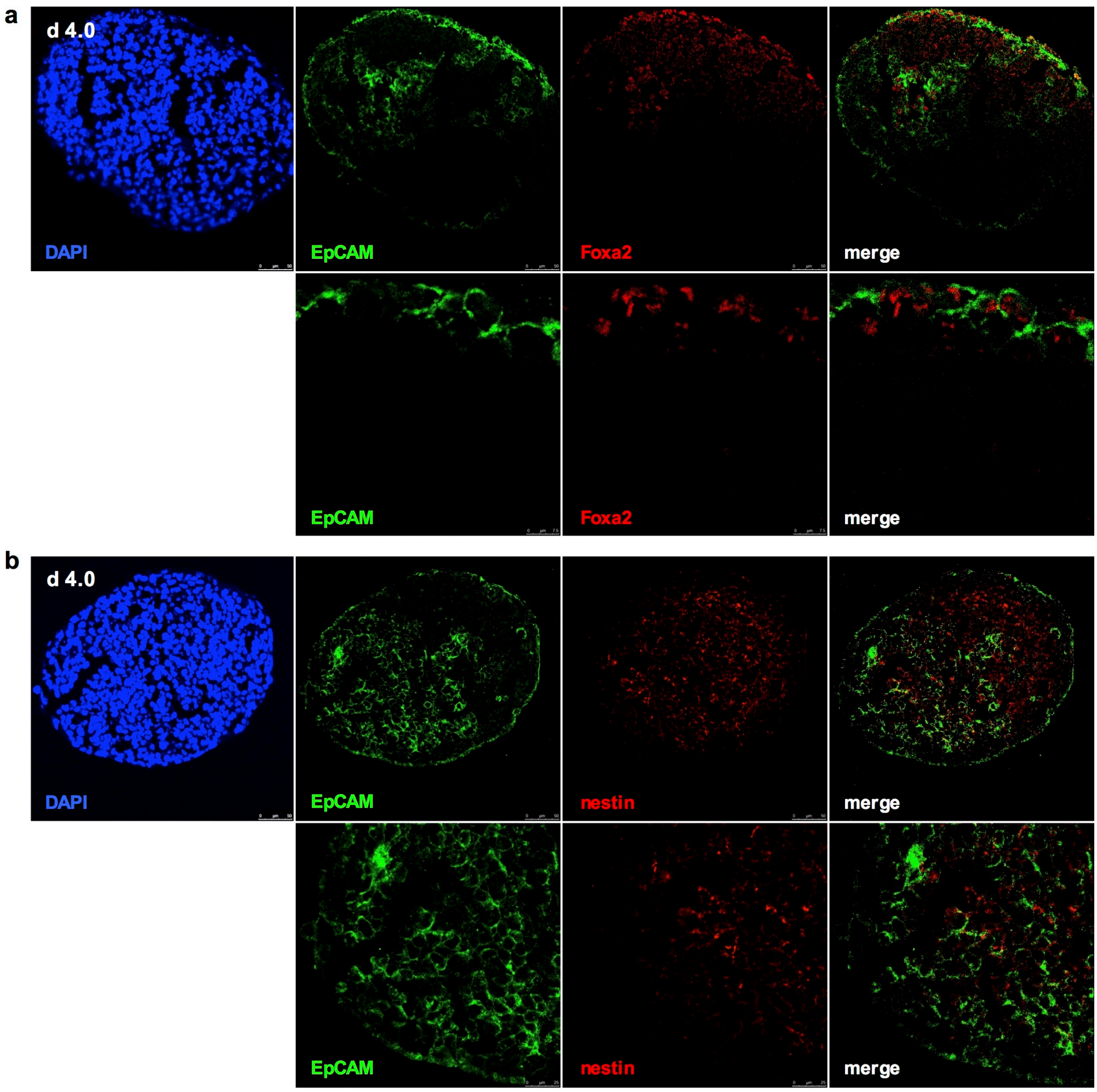

Supplementary Figure 5. EpCAM, Foxa2, and nestin expression in E14TG2α EB

(**a**) EpCAM/Foxa2 and (**b**) EpCAM/nestin protein expression were assessed by double-immunofluorescence staining and confocal laser scanning microscopy in E14TG2α EB at day 4 of spontaneous differentiation. Blue: DNA (DAPI); green: EpCAM; red: Foxa2 (a) and nestin (b). Shown are representative results from two independent experiments with multiple EBs.

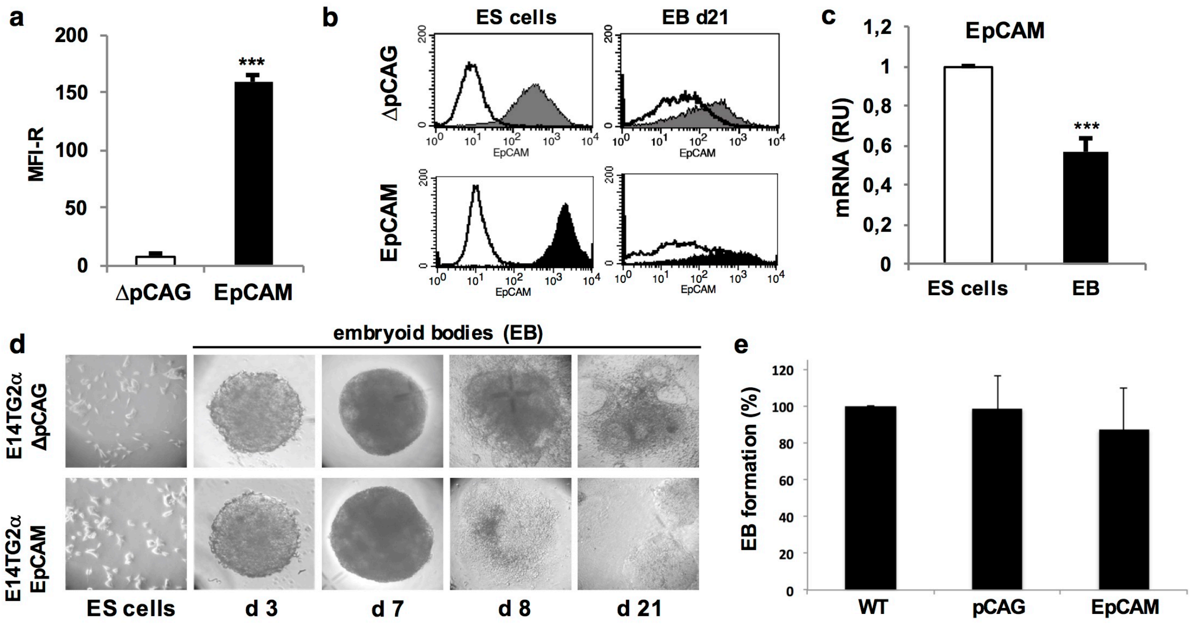

Supplementary Figure 6. EpCAM over-expression in ESC

(a) Mean fluorescence intensity ratio (MFI-R) of EpCAM divided by isotype control values in pCAG and EpCAM-expressing E14TG2α ESC (n=3 independent experiments). **(b)** Cell surface expression of EpCAM in pCAG and EpCAM expressing E14TG2α ESC under pluripotency and at day 21 of spontaneous differentiation. **(c)** EpCAM mRNA expression in pluripotent E14TG2 ESCs (d0) and EB (d2-14) stably transfected with pCAG-EpCAM expression plasmid (n=3 independent experiments). **(d)** Representative pictures of pCAG and EpCAM expressing E14TG2α ESC under pluripotency and as EB. **(e)** Percent of EB formation of wild-type (WT), pCAG and EpCAM expressing E14TG2α ESC (n=3 independent experiments). Mean ±SEM; Student´s T-test (n=2 groups) or One-Way ANOVA (n≥3 groups), *** $p<0.001$.

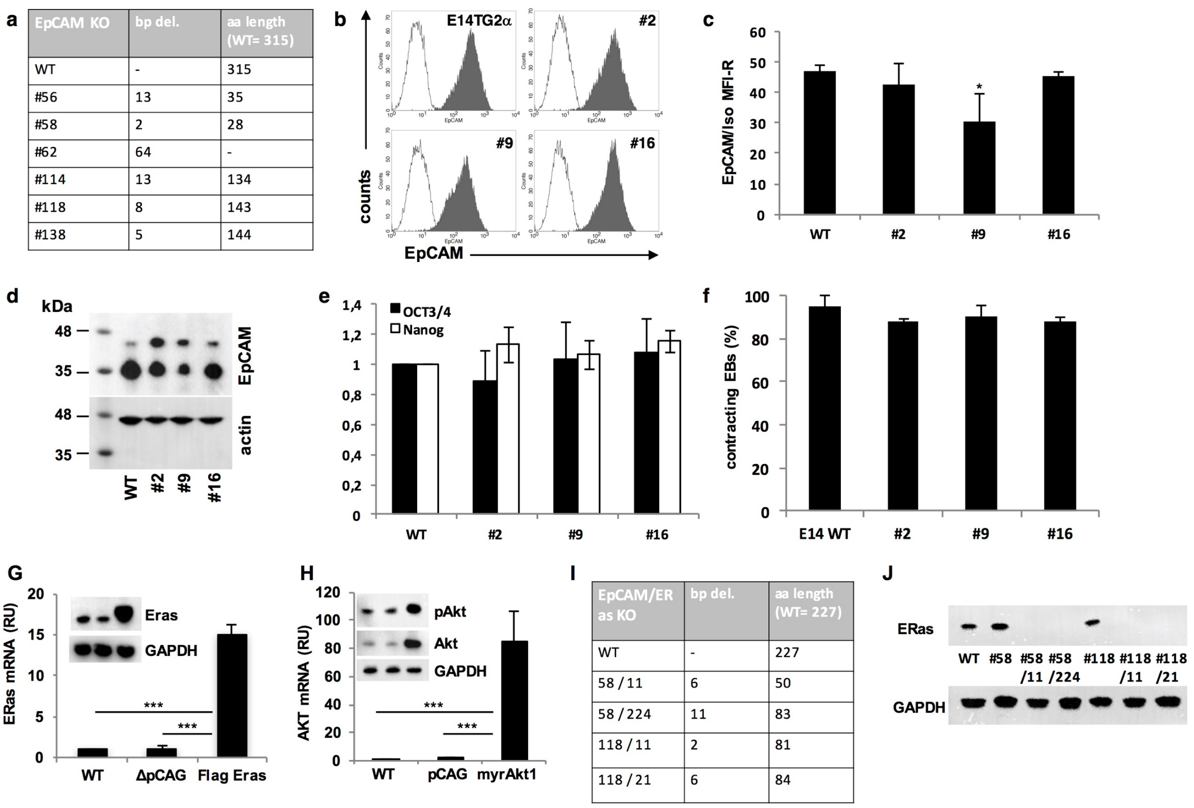

EpCAM KO	bp del.	aa length (WT= 315)
WT	-	315
#56	13	35
#58	2	28
#62	64	-
#114	13	134
#118	8	143
#138	5	144

EpCAM/ERas KO	bp del.	aa length (WT= 227)
WT	-	227
58 / 11	6	50
58 / 224	11	83
118 / 11	2	81
118 / 21	6	84

Supplementary Figure 7. **EpCAM and ERas knockout in ESC**

(**a and i**) Base pair deletions and resulting amino acid length of the putative EpCAM and ERas protein in single *EPCAM* and double *EPCAM/ERAS* CRISPR-Cas9 Cas9 knockout clones. (**b**) Cell surface expression of EpCAM in wild-type (WT) and CRISPR-Cas9 clones #2, #9 and #16 under pluripotency conditions. Shown are representative diagrams of flow cytometry measurements. (**c**) Quantification of cell surface expression of EpCAM in wild-type (WT) and CRISPR-Cas9 clones #2, #9 and #16 under pluripotency conditions (n=3 independent experiments). (**d**) EpCAM expression was assessed in whole cell lysates of E14TG2α ESC and CRISPR-Cas9 knockout clones #2, #9 and #16 under pluripotency conditions. Equal loading was demonstrated upon staining of actin. (**e**) *Oct3/4* and *NANOG* mRNA expression measured by quantitative PCR in pluripotent (ES cells) and differentiated transfectants (n=3 independent experiments). (**f**) Percent contracting EB of wild-type E14TG2α ESC (WT) and *EPCAM* knockout clones #2, #9 and #16 at day 10 of spontaneous differentiation (n=3 independent experiments). **(g)** ERas mRNA expression measured by quantitative PCR in wild-type E14TG2α ESC, pCAG controls and Flag-ERas expressing transfectants (n=3 independent experiments). ERas protein expression assessed by immunobloting in wild-type E14TG2α ESC, pCAG controls and Flag-ERas expressing transfectants (n=3 independent experiments). Immunoblot gels are depicted as inserts within mRNA expression graph. **(h)** Myristoylated AKT (myr-AKT) mRNA expression measured by quantitative PCR in wild-type E14TG2α ESC, pCAG controls and myrAKT expressing transfectants (n=3 independent

experiments). AKT and phosphorylated AKT protein expression assessed by immunobloting in wild-type E14TG2α ESC, pCAG controls and myrAKT expressing transfectants (n=3 independent experiments). Immunoblot gels are depicted as inserts within mRNA expression graph. (**j**) ERas protein expression assessed by immunobloting in single EpCAM knockout clones #58 and #118 and in double EpCAM/ERas knockout clones #58/11, #58/224, #188/11 and #118/21 (n=3 independent experiments). Mean ±SEM; Student´s T-test (n=2 groups) or One-Way ANOVA ($n \geq 3$ groups); *** $p<0.001$.

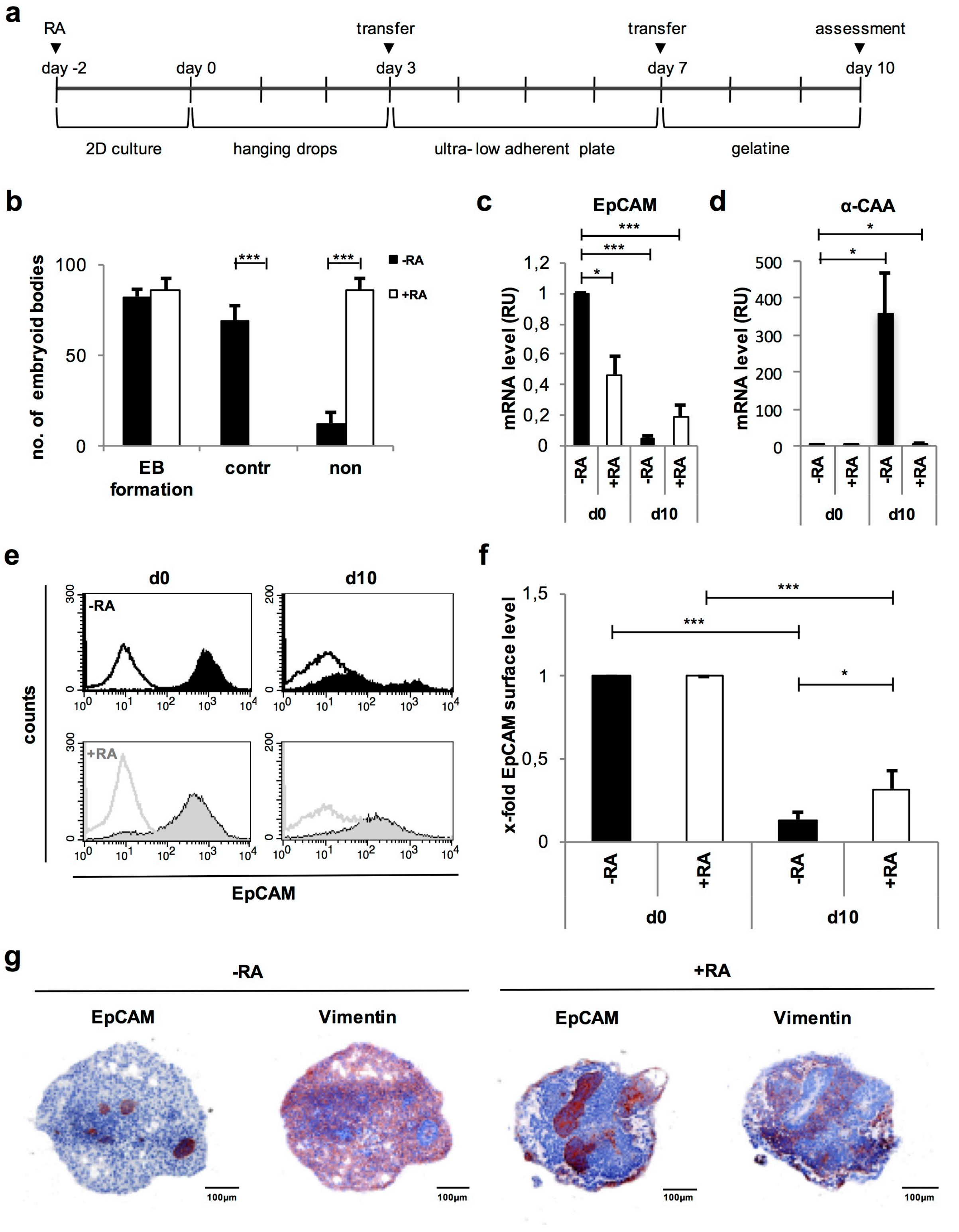

Supplementary Figure 8. EpCAM expression under cardiomyocyte suppressive conditions

(**a**) Schematic illustration of RA treatment and EB generation. (**b**) Percent EB formation and contraction in RA-treated and control conditions. +RA; open bars; -RA; black bars (n=3 independent experiments). (**c-d**) EpCAM and α-CAA mRNA expression in control and RA-treated E14TG2α ESCs after two days of treatment (d0) and in EBs at d10 of differentiation

(n=3 independent experiments). (**e**) Cell surface expression of EpCAM in control and RA-treated ESC at day 0 and 10 (n=3 independent experiments). **(f)** Quantification of cell surface expression of EpCAM in control and RA-treated ESC at day 0 and 10 (n=3 independent experiments). (**g**) EpCAM and vimentin protein expression in controls and RA-treated EB.
Mean ±SEM; Student´s T-test; *p<0.05, ** p<0.001, *** p<0.0001.

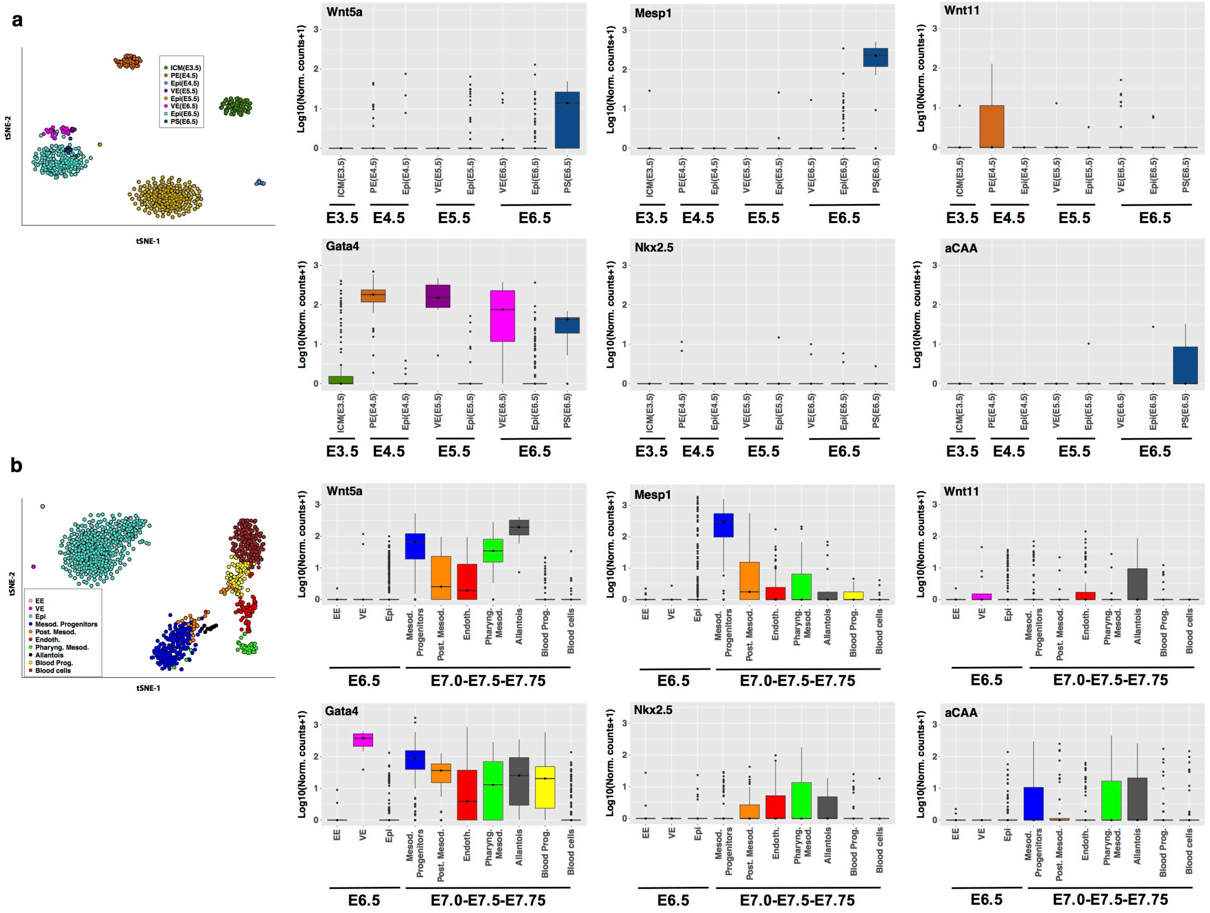

Supplementary Figure 9. Wnt5a, Mesp1, Wnt11, Gata4, Nkx2.5 and α-CAA in early gastrulation

Transcript levels of Wnt5a, Mesp1, Wnt11, Gata4, Nkx2.5, and α-CAA were assessed from RNA-sequencing datasets generated from single cells at day E3.5-6.5 (**a**) and E6.5-7.75 (**b**). We show a visualization of the datasets with t-distributed stochastic neighbor embedding (upper left panels), by highlighting the clusters defined in the original publications. Transcript levels are depicted as box-whisker plots with $\log_{10}$ normalised counts (adding a pseudocount of 1), and colored according to cell type. See Figure 1 and Methods for additional details.

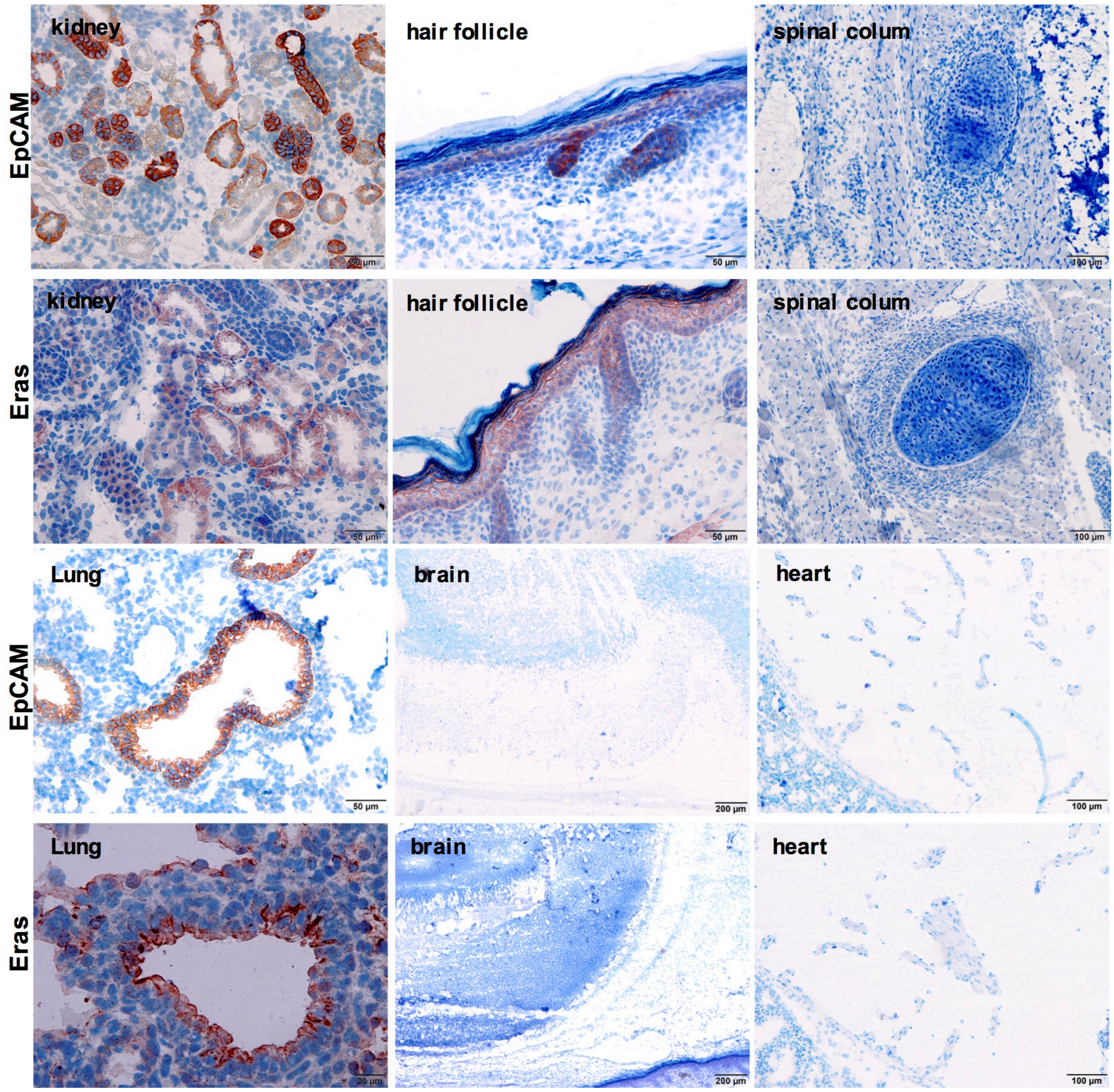

Supplementary Figure 10. EpCAM and ERas expression in E18.5 murine embryos

The expression of EpCAM and ERas is depicted as immunochemistry staining in E18.5 C57BL-6 mouse embryos. Shown are magnifications of kidney, hair follicles, spinal column, lung, brain, and heart for both antigens (brown staining).

Rich media file legend

Video 1:

Contraction of cardiomyocytes after spontaneous differentiation of murine embryonic stem cells (ESCs) in three-dimensional embryoid bodies (EB). Murine ESCs were spontaneously differentiated in hanging drops, transferred to low-adherence plates at day 7, and contraction of differentiated cardiomyocytes monitored at day 10. Shown is a representative contractile area of one EB.
